# Proteomics and liquid biopsy characterization of human EMT-related metastasis in colorectal cancer

**DOI:** 10.3389/fonc.2022.790096

**Published:** 2022-09-28

**Authors:** Mao-Sen Huang, Li-Hua Fu, Hao-Chao Yan, Lin-Yao Cheng, Hai-Ming Ru, Si Mo, Chun-Yin Wei, Dai-Mou Li, Xian-Wei Mo, Wei-Zhong Tang, Lin-Hai Yan

**Affiliations:** ^1^ Department of Gastrointestinal Surgery, Guangxi Medical University Cancer Hospital, Nanning, China; ^2^ Department of Gastrointestinal Surgery, Guangxi Clinical Research Center for Colorectal Cancer, Nanning, China; ^3^ Department of Gastrointestinal Surgery, Guangxi Key Laboratory of Colorectal Cancer Prevention and Treatment, Nanning, China

**Keywords:** epithelial-mesenchymal transition, proteomics, liquid biopsy, MCRC, CTC (circulation tumor cells)

## Abstract

Tumor cells undergo epithelial-mesenchymal transition (EMT), however, there is a room of disagreement in role of EMT heterogeneity to colorectal cancer metastasis (mCRC) evolution. To uncover new EMT-related metastasis proteins and pathways, we addressed the EMT status in colorectal cancer liver metastasis patient-derived CTCs to identify proteins that promote their distant metastasis. And then, we performed a comparative proteomic analysis in matched pairs of primary tumor tissues, adjacent mucosa tissues and liver metastatic tissues. By integrative analysis we show that, unstable Epithelial/Mesenchymal (E/M)-type CTCs had the strongest liver metastases formation ability and the proportion of E/M-type CTCs correlated with distant metastases. Using an optimized proteomic workflow including data independent acquisition (DIA) and parallel reaction monitoring (PRM), we identified novel EMT-related protein cluster (GNG2, COL6A1, COL6A2, DCN, COL6A3, LAMB2, TNXB, CAVIN1) and well-described (ERBB2) core protein level changes in EMT-related metastasis progression, and the proteomic data indicate ERBB2, COL6A1 and CAVIN1 are promising EMT-related metastatic biomarker candidates. This study contributes to our understanding of the role that EMT plays in CRC metastasis and identifies heterogeneous EMT phenotypes as a key piece for tumor progression and prognosis. We further propose that therapies targeting this aggressive subset (E/M-type) of CTCs and related protein may be worthy of exploration as potential suppressors of metastatic evolution.

## Introduction

Colorectal cancer (CRC) is the third most prevalent cancer worldwide, and the second leading cause of cancer-related mortality (1). The major cause of death in CRC patients is metastasis, and the primary and most common site of metastatic CRC (mCRC) is the liver (2–4). Early detection can improve clinical outcomes for CRC patients with liver metastases.

Circulating tumor cells (CTCs) are the direct cause of cancer metastasis, and specific types of CTCs give rise to metastatic foci under different conditions. In the process of colorectal tumor metastasis, tumor cell invasion, intravascular and extravasation are accompanied, eventually leading to distant organ colonization of CTCs. In addition, the CTC load correlates with poor progression free survival in mCRC (5–7). Epithelial-Mesenchymal Transition (EMT), a reversible biological program wherein epithelial cells gradually transform into the highly invasive stromal cells, is a key event in tumor metastasis (8). CTCs enhance migration and aggressiveness through EMT, and interstitial CTCs are a biomarker of cancer progression (9–11). There are also three subtypes of CTCs undergoing EMT process. Epithelial (E), Mesenchymal (M) and E/M (Epithelial/Mesenchymal) CTCs. Furthermore, the predominantly epithelial CTC subtypes (E and E/M) have stronger metastatic and proliferative abilities (12). However, the prognostic impact of different CTC phenotypes is still ambiguous.

Proteomics-driven precision medicine (PDPM) relies on the detection of very low levels of protein biomarkers in the early stages of cancer through highly sensitive proteomics, i.e., Qualitative, and quantitative analysis of all proteins in a biological unit. The current challenges in proteomics technology are the analytical speed, proteome coverage depth and quality of data analysis. Data Independent Acquisition (DIA) system can simultaneously analyze the proteomes of multiple samples, and therefore obviate the above limitations and scan more data without losing low abundance proteins (13). In addition, parallel reaction monitoring (PRM) mass spectrometry is a high throughput technology that can simultaneously verify dozens of proteins. The samples can be directly detected by mass spectrometry without the need for specific antibodies, which can improve the accuracy and success rate of verification (14). Combining non-labeled target protein screening by DIA and further verification by PRM is a viable strategy for identifying novel disease-related biomarkers (15, 16).

In the present study, we found that the transient epithelial/mesenchymal (E/M)-type CTCs from mCRC patients have the strongest metastatic abilities. Subsequently, we used the comparative proteomic approach to identify novel biomarkers and EMT-related pathways in the matched pairs of primary tumor tissues, adjacent mucosal tissues, and liver metastatic tissues from symptomatic early T staging (T_2_N_x_M_1_) mCRC patients. The proteomics data was analyzed by DIA and PRM. Our findings indicate that different CTC subpopulations stratified based on the EMT phenotype and related proteomics should be considered as targets for multimodal therapy.

## Materials and methods

### Samples and patient collection

Circulating tumor cells(CTCs)from 100 patients with CRC were analyzed. We studied the difference between non-metastatic patients and metastatic patients. Our 100 patients already included 70 mCRC and 30 non-metastatic colorectal cancer patients. In addition, patients with colorectal cancer included in our study were rigorously screened. Inclusion criteria: 1) first-diagnosed patients; 2) colorectal cancer was confirmed by pathological biopsy of colorectal endoscope; 3) CT or PECT of colorectal cancer patients with liver metastases clearly showed liver metastases. We selected suitable patients for proteomic studies who were diagnosed with colorectal cancer by preoperative colonoscopy biopsy and had obvious liver metastases on preoperative imaging. 9 mCRC samples for which matched pairs of primary tumor tissues, adjacent mucosa tissues and liver metastases tissues were evaluated in the study (online supplementary methods).

### Circulating tumor cells isolation and identification

70 histologically confirmed metastatic colorectal cancer and 30 non-metastatic colorectal cancer (total = 100) that had matched pairs of tissue and peripheral blood samples for CTC analysis using a Canpatrol^®^ system were employed. For further details, refer to the online supplementary methods section. We capture the fluorescent markers of EMT morphological transformation of CTC formed in the process of transfer. There are three forms respectively, and the detectable markers of each form are different. Canpatrol system uses EpCAM, CK8, CK18 and CK19 to mark E-type CTC, twist and vimentin to mark M-type CTC, and EpCAM, CK8, CK18, CK19, twist and vimentin to mark E/M-type CTC. We need to capture the fluorescence intensity of the whole image marker to distinguish the subtypes of EMT.

### CTCs isolation and identification

Canpatrol^®^ system (SurExam Bio-Tech, China) be uesd for rapid, size-based capture of CTCs from peripheral blood (PB). CTCs separation, enrichment and classification identification of two parts, CTC enrichment and multiple RNA *in situ* analysis and detection.

### CTCs enrichment

According to the CTC enrichment technology of ISET, using a calibrated membrane with 8uM diameter pores and multiplex mRNA *in situ* hybridization (ISH) assay to identify and classify CTCs, the optimized enrichment technique is more effective for CTC isolation and characterization. 5ml peripheral blood of patients were collected in EDTA anticoagulated tube, then erythrocytes were removed by a red blood cell lysis buffer (154 mM NH_4_Cl, 10 mM KHCO_3_ and 0.1 mM EDTA). And the cells were transferred to the filtration tube after resuspension in PBS containing 4% formaldehyde for 5 minutes. Then the pump valve was switched on to reach at least 0.08MPa and the manifold vacuum plate valve was then switched on to fulfill filtration.

### Multiple RNA *in situ* analysis and detection

To help us distinguish epithelial, mesenchymal and hybrid CTCs. On the membrane of the 24-well plate, cells were treated with a protease before hybridization with indicated capture probe specific for EpCAM, CK8/18/19, vimentin, twist, and CD45 (**Supplementary Table 1**). After incubation at 42°C for 2 h, cells were washed with buffer to remove the unbound probes. Then cells were incubated with preamplifier solution [30% horse serum, 1.5% sodium dodecyl sulfate, 3mM Tris-HCl (pH 8.0) and 0.5 fmol of preamplifier; the sequences are shown in **Supplementary Table 2**] at 42°C for 2 h for the purpose of signal amplification. The membranes were washed with 1000 μl of wash buffer (0.1 × SSC), and then incubated with 100 μl of amplifier solution [30% horse serum, 1.5% sodium dodecyl sulfate, 3mM Tris-HCl (pH 8.0) and 1 fmol of amplifier; the sequences are shown in **Supplementary Table 2**]. Fluorescently labeled probes, which had been conjugated with fluorescent dyes Alexa Fluor 594 (for the epithelial biomarkers EpCAM and CK8/18/19), Alexa Fluor 488 (for the mesenchymal biomarkers vimentin and twist), Alexa Fluor 750(for CD45), Alexa Fluor 647 (CD133), were added and incubated at 42°C for 2 min. After staining with DAPI, cells were analyzed with a fluorescence microscope (Olympus BX53, Tokyo, Japan). Red and green fluorescence signal points represent the expression of epithelial and interstitial genes on CTC

## Data independent acquisition and parallel reaction monitoring

### Data independent acquisition

#### Sample preparation

The frozen sample was added to liquid nitrogen for grinding and then transferred to a 1.5mL centrifuge tube. Then 300 L sample lysis buffer and PMSF were added to bring the final concentration to 1mM. Ultrasonic crushing was placed on the ice, with power of 80W. Ultrasonic and shutdown were alternated for 1 second each, a total of 3min. Centrifuge at 12000×g for 10min at room temperature, take the supernatant, and repeat. The supernatant is the total protein solution of the sample. The protein concentration was measured and stored at -80°C after separation.

### Protein concentration determination

The protein concentration was determined by BCA method, and the color developing solution was prepared by buffer A: Buffer B=50:1(V/V). The protein solution to be measured was diluted to the working range of the standard curve by adding ultra-pure water. BSA standard protein solution was added to 96-well plates at a concentration gradient of 0,1,2,4,8,12,16,20 L. Ultraportable water was added to each well to replenish the volume to 20 L. A 2 L protein solution was added to a 96-well plate with three replicates per sample. The volume was also increased to 20 L.A 200 L pre-prepared chromogenic solution was added and reacted at 37°C. After 30 minutes, the absorbance value (wavelength 562nm) was measured using a microplate analyzer, and the protein concentration value was calculated according to the known concentration and absorbance value of the standard protein solution.

### SDS-PAGE electrophoresis

10 g protein was extracted from each sample and separated by 12% SDS-PAGE. Then the samples were dyed by Coomasilan staining with reference to the experimental method of Candiano et al. That is, fixed for 2h, dyed for 12h, washed until the background was clear, and then scanned in full color with ImageScanner scanner with an optical density value of 300 dpi

### Trypsin hydrolysis

According to the determined protein concentration, 50 g of protein was taken from each sample and diluted with lysis buffer to the same concentration and volume. DTT was then added to make the final concentration of DTT 4.5mm, mixed well, and incubated at 55°C. After 30 minutes cool on the ice to room temperature. The corresponding volume of iodoacetamide was added to make the final concentration 9mM, which was thoroughly mixed and placed at room temperature and away from light for 15min. Then 6 times of the volume of precooled acetone in the above system to precipitate the protein, and then place it at - 20°C for more than 4 hours or overnight. The precipitation was collected by centrifugation at 8000×g for 10 minutes at 4°C, and the acetone was volatilized for 2-3 minutes.100 L TEAB2 was redissolved and precipitated. 1mg/mL trypsin-TpCK of 1/50 sample mass was added and digested overnight at 37°C.Add phosphoric acid and adjust PH to 3 or so to stop the enzymatic hydrolysis reaction.

### Desalination

After enzymatic hydrolysis, the peptides were desalted by RP-C18 spE column: Methanol 1mL, 90% acetonitrile-water 1mL and water (containing 0.1%TFA) were rinsed once respectively. And the samples were loaded on the column 3 times. Then the column was washed by 0.1%TFA/H2O 3 times. Finally, the peptides were eluted with 90% ACN/H2O (containing 0.1%TFA) 3 times, vacuum-dried and redissolved with 60 L 0.1% formic-water.

### LC-MS/MS high resolution mass spectrometry

#### RPLC analysis

RP separation was performed on an 1100 HPLC System (Agilent) using an Agilent Zorbax Extend RP column (5 μm, 150 mm × 2.1 mm). Mobile phases A (2% acetonitrile in HPLC water) and B (90% acetonitrile in HPLC water) were used for RP gradient. The solvent gradient was set as follows: 0~10 min, 98% A; 10~10.01 min, 98%~95% A; 10.01~37 min, 95%~80% A; 37~48 min, 80~60% A; 48~48.01 min, 60~10% A; 48.01~58 min, 10% A; 58~58.01 min, 10~98% A; 58.01~63 min, 98% A. Tryptic peptides were separated at an fluent flow rate of 250μL/min and monitored at 210 nm. After 10 minutes, the eluent was collected at one-minute intervals into the no. 1-10 centrifuge tube, and the fractions of the 10 components were recycled in sequence. Vacuum freeze-drying was performed and cryopreserved for mass spectrometry.

#### Mass spectrometry analysis

All analyses were performed by a Q-Exactive HF mass spectrometer (Thermo, USA) equipped with a Nanospray Flex source (Thermo, USA). Samples were loaded and separated by a C18 column (50 cm × 75 μm) on an EASY-nLCTM 1200 system (Thermo, USA). The flow rate was 300 nL/min and linear gradient was 90 min (0~82min, 5-44%B; 82~84 min, 44-90% B; 84~90 min, 90% B; mobile phase A = 0.1% FA in water and B = 0.1% FA in 80%ACN).

#### DDA

The scanning mass spectrometry was set to have a scanning mass range of 350-1650m/z, a resolution of 120,000, and an automatic gain control value (AGC) of 3e6. The acquisition of MS/MS atlas was completed by high-energy collision pyrolysis with an energy of 27, with a resolution of 30000 and an AGC control of 2e5. The Q Exactive HF dynamic exclusion was set for 40.0s and run under positive mode.

#### DIA

Full MS scans were acquired in the mass range of 350 - 1250 m/z with a mass resolution of 120000 and the AGC target value was set at 3e6. The 32 acquisition windows in MS were fragmented with higher-energy collisional dissociation (HCD) with collision energy of 28 and each acquisition window has 26 m/z. MS/MS spectra were obtained with a resolution of 30000 with an AGC target of 1e6 and a max injection time is set to auto and run under positive mode.

### Database search

Spectronaut was used to search all of the raw data thoroughly against the sample protein database. Database search was performed with Trypsin digestion specificity. Alkylation on cysteine was considered as fixed modifications in the database searching. Protein, peptide and PSM’s false discovery rate (FDR) all set to 0.01. For DIA data, the quantification FDR also set to 0.05. Quantity MS-level was set at MS2.

### Parallel reaction monitoring

Reversed-phase high performance liquid chromatography (RP-HPLC)

RP separation was performed on an 1100 HPLC System (Agilent) using an Agilent Zorbax Extend RP column (5 μm, 150 mm×2.1 mm). Mobile phases A (2% acetonitrile in HPLC water) and B (90% acetonitrile in HPLC water) were used for RP gradient. The solvent gradient was set as follows: 0-10 min, 98% A; 10-10.01 min, 98%-95% A; 10.01-37 min, 95%-80% A; 37-48 min, 80-60% A; 48-48.01 min, 60-10% A; 48.01-58 min, 10% A; 58-58.01 min, 10-98% A; 58.01-63 min, 98% A. Tryptic peptides were separated at an fluent flow rate of 250μL/min and monitored at 210 nm. Samples were collected for 10-50 minutes, and eluent was collected in centrifugal tube 1-10 every minute in turn. Samples were recycled in this order until the end of gradient. The separated peptides were lyophilized for mass spectrometry.

### PRM pre-scan

The sample used for the prescan was the equivalent mix of all samples to correct for the retention time of the peptide segment.

(1) Pre-sweep method setting

When SpectroDive software loads the target protein list, matches the corresponding Library (i.e. the above DDA Library), calculates the mass-charge ratio of the theoretical peptide sequence of the target protein, and unique peptides that satisfy the following conditions are retained: ProteinGroup specific or proteoctypic specific; Miss cut to zero; Immutable modification; Charge number: 2-3.

(2) Export the above list and set up a mix pre-scan on the mass spectrometry method.

(3) The SpectrDive software loads the pre-scanned RAW data and corrects the retention time.

(4) Set the scheduled method and export.

PRM mode detection

(1)The chromatographic conditions

ACN-H2O-FA was used for both mobile phase A and B, and the proportions were 0:10, 0:0.1, V/V/V and 80:20:0.1, v/V/V, respectively. The velocity is set to300 nL/min. Gradient elution condition is set as 0~ 78min, 2-40% B; 78~80 min, 40-90% B; 80~90 min, 90% B.

(2)Mass spectrometry conditions:

The exported raw data list was imported into the Inclusion List of the Xcalibur-PRM method editing module with MS2 resolution set to 17500, the separation window set to 1.4m/z, and the AGC set to 1e5.

### Statistical analysis

We performed all statistical analyses using SPSS version 17.0 statistical software (SPSS, Chicago, USA). The measurement data were presented as the mean ± standard error of mean (S.E.M), and statistical significance was determined by t-tests. The relationships between gene levels and variables were analyzed by conducting the Spearman’s, Pearson’s, and linear regression analyses. Statistical significance was calculated using Origin 8.0 software programs (OriginLab, USA) and was considered at p-value < 0.05. GraphPad Prism 8 software (GraphPad Software, Inc., San Diegop>.

## Results

### Patient characteristics

**Figure 1:**
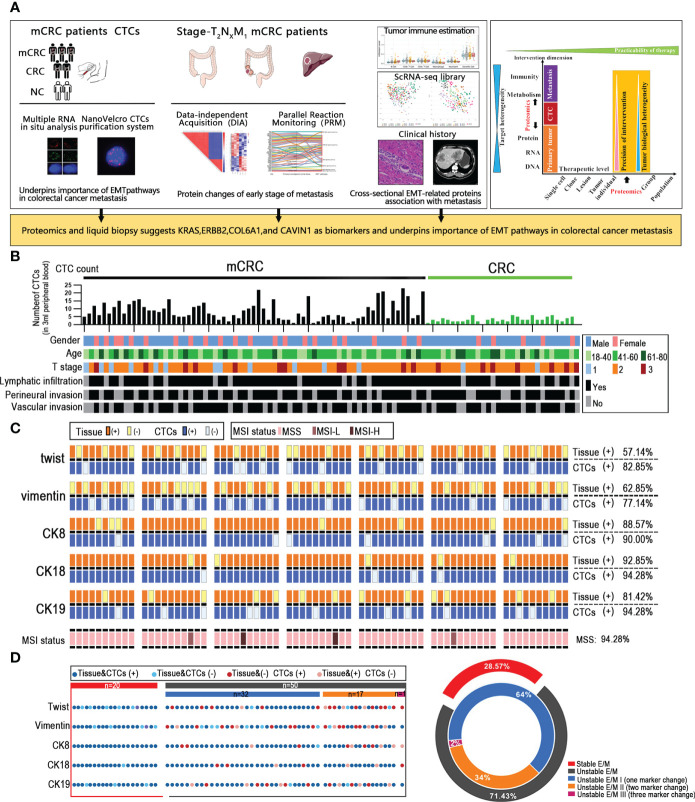
Unstable tumor and CTCs EMT state correlate with the clinical outcome of mCRC patients. **(A)** A scheme showing experiments and integrated analyses which were performed. **(B)** CTCs profile and associated clinicopathologic features of all the mCRC patients and CRC patients. **(C)** The landscape of EMT molecular alterations detected in CTCs and the alterations detected in paired tissue samples is presented. **(D)** Shown are proportions of stability molecular expression CTCs in individual patients; red: stable, gray: instability.

Most CRC patient exhibit metastases at the initial diagnosis due to the occult nature of the process. To uncover underlying mechanisms involved in metastasis at early stage and develop a clinically practicable therapy, we comprehensively investigated the CRC with metastasis across multiple dimensions (**Figure 1A**). The CTC data of 70 metastatic colorectal cancer patients, 30 non-metastatic colorectal cancer patients and 10 healthy controls, and the proteomic data of 3 CRC patients with liver metastasis (T_2_N_x_M_1_) were collected. After screening for the CTC subtype with strongest correlation to distant metastasis, we selected 3 mCRC patients at early T stage (T_2_) with distant metastasis, and analyzed the proteomics data of their primary tumor, normal intestinal epithelium, and metastatic liver tissues. Finally, the CancerSEA and TIMER database were used to analyze the single cell function and immune invasion degree of these selected possible influencing factors and predicted proteins The primary and metastatic lesions of all patients who underwent CTC detection were assessed by HE staining, lymphocyte immunophenotyping and TUNEL staining, and the expression levels of MLH2, MSH6, PMS2 and MLH1 were also analyzed (**Figure S1A**).

### Unstable tumor and CTCs EMT state correlate with the clinical outcome of mCRC patients

Given that CTCs are the source of distant metastasis, we next assessed the correlation between the EMT-related molecular alterations in primary tumor cells and CTCs. MSS/MSI status of colorectal cancer patients is closely related to poor prognostic outcomes. To this end, we analyzed the EMT and microsatellite instability (MSI) status-related genes in a cohort of 70 mCRC patients with liver metastases and 30 non-metastatic CRC patients. The expression of CK7, CK20, vimentin, β-catenin, Ki67, S100, P53, MLH1, PMS2, MSH2 and MSH6 in the tumor tissues were evaluated by IHC. The Canpatrol system was used to determine the expression levels of CK8, CK18, CK19, vimentin and Twist in the CTCs. As is evident from the expression of EMT proteins in CTCs and the clinical characteristics of the patients, the CTC load was higher in patients with definite metastasis versus the non-metastatic patients, which is also consistent with previous studies (10). However, the number of CTCs was not correlated to the extent of metastasis, lymphocyte infiltration, vascular invasion, and nerve invasion in the primary tumor, and therefore cannot totally reflect the metastasis status. CK8 and CK18 showed the highest expression levels in both the CRC tissues and CTCs, with considerable heterogeneity. In addition, 60 (57.14%) tissue samples and 62 (82.85%) CTC samples expressed Twist. Forty-five patients expressed Twist in tumor tissues and CTCs, whereas both were negative in 9 patients. Vimentin was expressed in 50 (62.85%) tissues and 57 (77.14%) CTCs samples, and both were respectively positive and negative in 39 and 11 patients. Furthermore, 66 (94.28%) tissues were microsatellite stable (MSS), and 2 patients (2.85%) had respectively low-frequency (MSI-L) and high-frequency (MSI-H) microsatellite instability. In summary, the expression levels of EMT-related genes were overall consistent between tumor tissues and CTCs, and CTCs profiling may provide valuable insights into the EMT status of CRC tumors. Through the analysis by our data, since the MSI status does not affect EMT and the MSI-H patients account for only a small fraction of all mCRC patients, the MSI state need not be considered when analyzing EMT. Based on whether the core EMT molecules were expressed in both or neither tumor tissues and matched CTCs, the patients were stratified as stable and unstable E/M respectively (**Figures 1B, C**). 20/70 mCRC patients (28.57%) had stable E/M and 50 patients (71.43%) had unstable E/M. In addition, 32/50 patients with unstable E/M (64%) showed inconsistent expression of one biomarker, 17 patients (34%) expressed two biomarkers differentially between tissues and CTCs, and one patient (2%) showed inconsistent expression of three biomarkers (**Figure 1D** **)**.

### Hybrid epithelial/mesenchymal phenotype (E/M) CTCs contributes to liver metastasis of mCRC

**Figure 2:**
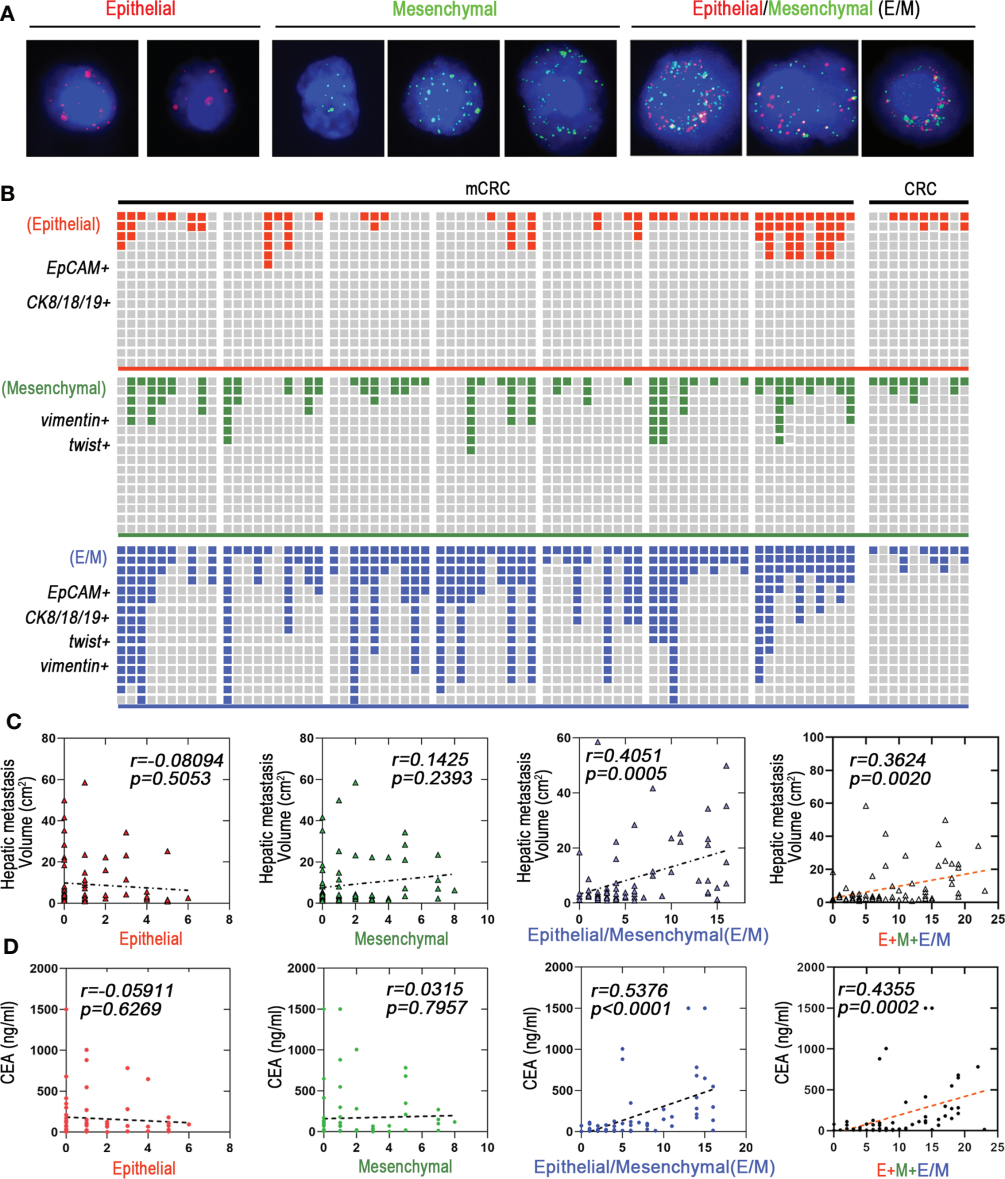
Hybrid epithelial/mesenchymal phenotype (E/M) CTCs contributes to liver metastasis of mCRC. **(A)** CTCs were detected by Canpatrol^®^ system (red: EpCAM, CK8, CK18, and CK19, green: vimentin and twist). DAPI (4′,6-diamidino-2-phenylindole). **(B)** Shown are proportions of E, M, and E/M phenotype CTCs in individual mCRC patients (n=70) and CRC patients (n=30). The x-axis represents each patient. The Y-axis represents the overall fluorescence intensity for each EMT type in each patient(Fluorescence intensity of Canpatrol system uses EpCAM, CK8, CK18 and CK19 to mark E-type CTC, twist and vimentin to mark M-type CTC, and EpCAM, CK8, CK18, CK19, twist and vimentin to mark E/M-type CTC. In different types of EMT subtypes of each patient, red, blue and green squares represent the proportion of fluorescence intensity of the patient in all patients). **(C)** E, M, and E/M phenotype CTCs to predict the liver metastatic tumor volume are depicted as a linear regression model. P value are indicated. **(D)** E, M, and E/M phenotype CTCs to predict the carcino-embryonic antigen (CEA) are depicted as a linear regression model. P value are indicated.

The immunophenotype of tumor cells change constantly during EMT, based on which they are classified as epithelial (E), predominantly epithelial/mesenchymal mixed (E/M), predominantly mesenchymal/epithelial mixed phenotype (M/E), and mesenchymal (M) (17). Based on our findings, we propose that cells undergoing EMT can be stratified into the stable EpCAM^+^CK8^+^CK18^+^CK19^+^ epithelial (E), stable vimentin^+^twist^+^ mesenchymal (M) and the transitioning unstable EpCAM^+^CK8^+^CK18^+^CK19^+^vimentin^+^twist^+^ epithelial/mesenchymal (E/M) phenotypes (**Figure 2A**). As shown in **Figure 2B**, E/M was detected in 63/70 (90%) mCRC patients and was the major CTC phenotype in this cohort, but was rarely seen among the CRC patients. In addition, 42 mCRC patients showed high frequency of the E/M phenotype (more than 3 biomarkers). Furthermore, the number of E/M CTCs correlated significantly with the size of liver metastases (r=0.4051; p=0.0005), whereas the E and M phenotypes did not show any correlation. In addition, the total CTC load was also significantly correlated with the size of metastatic nodules in patients with predominantly E/M phenotype (r=0.3624, p=0.002) compared to all phenotypes combined (r=0.3624, p=0.002; **Figure 2C**). Furthermore, the number of E/M CTCs and carcinoembryonic antigen (CEA) levels were significantly correlated (r=0.5376, p<0.0005) compared to all phenotypes combined (r=0.4355, p=0.0002; **Figure 2D;** **Figure S2F**). In conclusion, the EMT status of the CTCs is a reliable indicator of metastasis in CRC, and the number of unstable E/M types is a measure of the extent of metastasis.

### Mass spectrometric analysis about proteomics characterization of mCRC

The proteomes of paired primary tumor (Ca), normal para-tumor intestinal epithelium (P) and liver metastatic tissues (Liver) from 3 T_2_N_x_M_1_ mCRC patients were analyzed by DIA and PRM methods. All patients presented with superficial muscular, perineural and vascular invasion, which are indicative of the early stages of metastasis (**Figure S1B** **)**. Since T2 stage can progress to distant metastasis, the factors identified in these patients may be more accurate indicators of metastasis compared to those identified in T3 and T4 mCRC patients.

**Figure 3:**
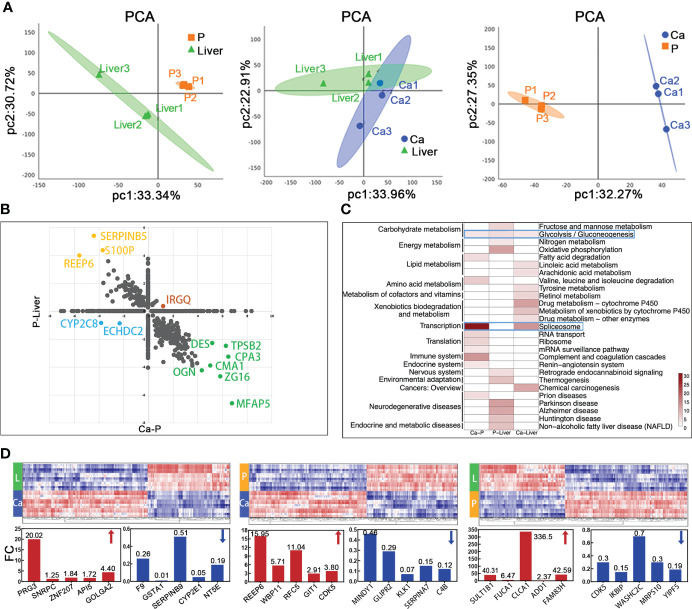
Mass spectrometric analysis about proteomics characterization of mCRC. **(A)** Principal-component analysis of protein expression in each group. **(B)** Merge of Ca-P and P-Liver proteomic data divide proteins in four groups. **(C)** The heatmap shows the significance of top 30 GO term being enriched by proteins in Ca-P, P-Liver and Ca-Liver. **(D)** Proteomic analysis reveals distinct protein expression patterns in paired tumor (Ca), para-tumor (P) and liver metastasis tissues (Liver). Heat maps of the differentially expressed proteins show clearly distinctive patterns of protein expression between disease groups.

LC-MS/MS identified 44,815 peptides encompassing 4,752 protein groups with a false discovery rate (FDR) of 1%, and the P value of most proteins was less than 0.01. Principal component analyses and hierarchical clustering analyses revealed distinct protein expression patterns between the paired Ca, P and Liver samples (**Figures 3A**; **S1C** **)**. Interestingly, the top five highly-expressed proteins in the same tissue were similar among patients. Furthermore, using fold change ≥ 1.5 and p-value<0.05 as the thresholds, we identified 347 differentially expressed proteins (DEPs) in P vs Liver, of which 211 were upregulated and 136 were downregulated in the former. Likewise, there were 261 DEPs in Ca samples relative to P, of which 82 and 179 proteins were respectively up- and downregulated. Finally, 144 DEPs were identified in Ca vs Liver samples, including 44 up-regulated proteins and 100 down-regulated proteins. (**Figure S1D**). Overall, 1262 proteins were differentially expressed in the Ca and Liver samples relative to P, and may be associated with metastasis. We classified these DEPs into the following four groups: (1) down-regulated in Ca vs P and up-regulated in P vs Liver, (2) up-regulated in both, (3) up-regulated in Ca vs P and down-regulated in P vs Liver, and (4) down-regulated in both (**Figure 3B**). KEGG pathway analysis revealed that the top 10 enriched pathways in each group were mostly related to metabolism, and the glycolysis/gluconeogenesis pathway was common to all groups, whereas the spliceosome pathway was most enriched among the DEPs between Ca and P samples (**Figure 3C**). Similar trends were observed with the top 50 enriched pathways as well (**Figure S1E**). Gene ontology (GO) enrichment analysis of the DEPs further revealed that the most significant biological processes were creatine biosynthesis and fructose biosynthesis, cellular components were collagen type IV trimer and exosomes, and molecular functions were 11-beta-hydroxysteroid dehydrogenase [NAD (P)] activity and signal recognition particle binding involved in CRC with liver metastasis (**Figure S1F**). As shown in **Figure 3D**, proliferation associated protein PRG3 and anti-apoptosis related protein SERPINB9 in Ca vs Liver group, and cyclin-dependent kinases CDKS up-regulated in both primary tumor and liver metastasis tissues. The top 50 DEPs showed significant correlation in each group (**Figure S1G**). Taken together, the identified DEPs are mostly involved in genetic information processing, metabolism, organismal systems, environmental information processing and human disease-related signaling pathways.

### EMT-related metastasis core kinesin and structural helper proteins

**Figure 4:**
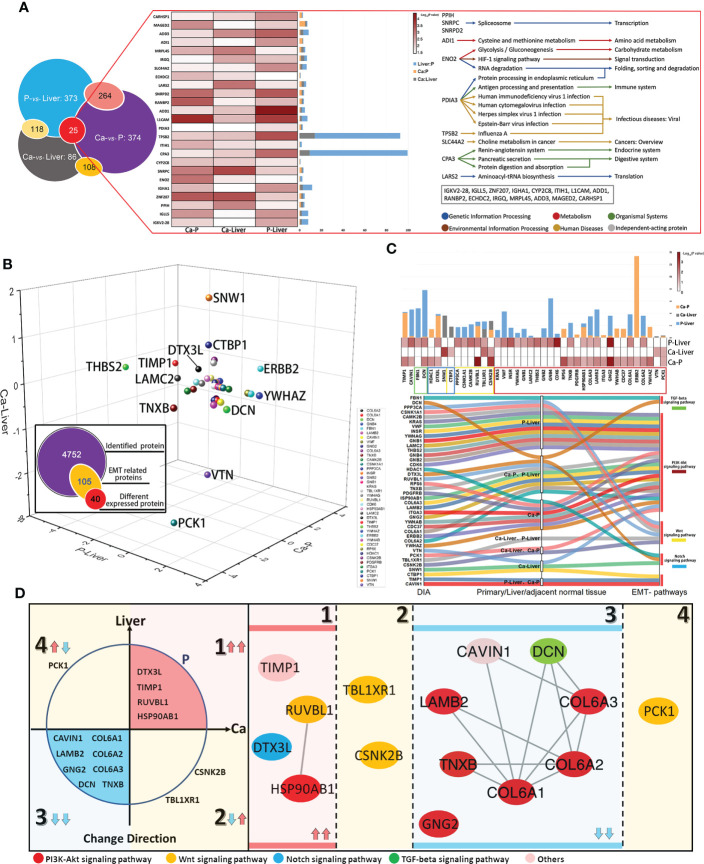
EMT-related metastasis core kinesin and structural helper proteins. **(A)** Relationship of differentially expressed proteins among Ca-Liver, Ca-P and P-Liver. Summary of 25 covariously expressed proteins in the three groups. **(B)** The change directions of 40 EMT-related differentially expressed proteins among Ca-Liver, Ca-P and P-Liver are shown in three-dimensional scatter plot. Log_2_ (FC) of protein levels in P-Liver (x axis), Ca-P (y axis) and Ca-Liver (z axis) are shown. **(C)** Integrated analysis of 40 EMT-related proteins. **(D)** 40 EMT-related Proteins can be divided into four groups: (1) upregulated in both Ca-P and Liver-P; (2) upregulated in Ca-Liver; (3) downregulated in both Ca-P and Liver-P; (4) downregulated in Ca-Liver.

Given the complex nature of metastasis, we performed network analysis of the proteomics data. Based on the above DIA analysis, 25 DEPs were identified in all comparison groups. ADI1, which is involved in cysteine and methionine metabolism, showed the most significant difference in expression levels between P and Liver groups. The largest fold change was exhibited by TPSB2, which regulates the influenza signaling pathway (**Figure 4A**). We further screened 105 EMT-related proteins from a total of 4752, of which 40 were differentially expressed in each group (**Figure 4B**). As shown in **Figure 4C**, the 40 DEPs common to all pairs were mainly enriched in PI3K-Akt signaling pathway, Wnt signaling pathway, Notch signaling pathway and TGF-beta signaling pathway, which are closely related to EMT. The differentially expressed EMT-related proteins were classified into four groups (**Figure 4D**): (1) up-regulated in Ca and Liver vs P, (2) up-regulated in Ca vs Liver, (3) down-regulated in Ca and Liver vs P, and (4) down-regulated in Ca vs Liver. Integration of the proteomics data with the 4 major signaling pathways showed that the Notch signaling pathway was activated and TGF-beta signaling pathway was suppressed in the primary and metastatic tumor tissues. PI3K-Akt signaling pathway is the enrichment of the most proteins. EMT-related proteins regulate DNA repair, angiogenesis, cell proliferation, apoptosis, glycolysis/gluconeogenesis, protein synthesis, cell cycle and proteolysis (**Figure S2A**). Based on these findings, we hypothesize that the network of structural helper proteins synergizes with the core kinesin during metastasis, and are potential early indicators of metastasis, as well as therapeutic targets.

### Clusters of EMT inferred by an integrated proteomic analysis

**Figure 5:**
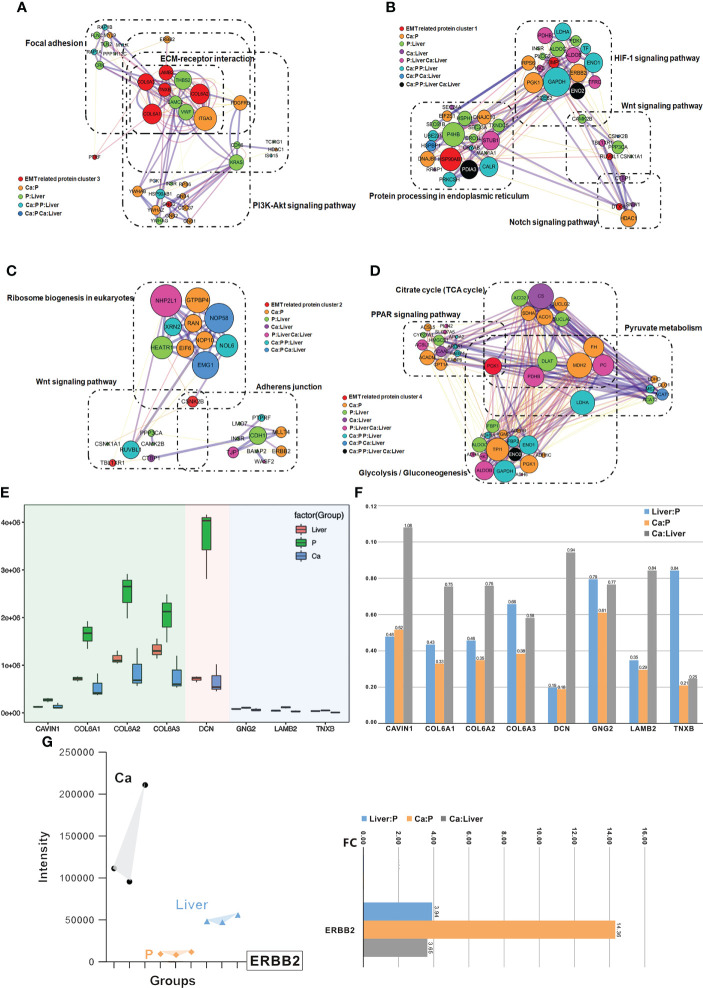
Clusters of EMT inferred by an integrated proteomic analysis. **(A)** All proteins in the pathway of differentially expressed proteins form the biggest EMT-related protein cluster. Interconnected network modules identified in some clusters. **(B)** All proteins in the pathway of differentially expressed proteins form EMT-related protein cluster (1). **(C)** All proteins in the pathway of differentially expressed proteins form EMT-related protein cluster (2). **(D)** All proteins in the pathway of differentially expressed proteins (PCK1) form EMT-related protein cluster (2), which majorly involved in glycolysis/gluconeogenesis, citrate cycle (TCA cycle), pyruvate metabolism and PPAR signaling pathway. **(E)** PRM analysis of EMT-related protein in the validation cohort. **(F)** The change of target protein expression. **(G)** The change of ERBB2 expression.

Protein-protein interaction (PPI) network was constructed with the EMT-related DEPs, which revealed four functional clusters **(** **Figure S2B** **)**. Cluster (iii) was mainly involved in ECM-receptor interaction, focal adhesion, PI3K-Akt signaling pathway and human papillomavirus infection signaling pathways (**Figure 5A**). Cluster (i) was the biggest and showed the strongest correlation with EMT, including proteins involved in Notch signaling pathway, HIF-1 signaling pathway, Wnt signaling pathway and protein processing in endoplasmic reticulum signaling pathway (**Figure 5B**). Furthermore, cluster (ii) consisted of proteins involved in Wnt signaling pathway, ribosome biogenesis in eukaryotes and adherens junction signaling pathway (**Figure 5C**), and cluster (iv) in glycolysis/gluconeogenesis, citrate cycle (TCA cycle), pyruvate metabolism and PPAR signaling pathway (**Figure 5D**). Interestingly, ERBB2 were the core proteins in only clusters (iii) and (i) **(** **Figure S2B** **)**. The main DEPs in cluster (iii) were GNG2, COL6A1, COL6A2, DCN, COL6A3, LAMB2, TNXB and CAVIN1, and may be most significantly correlated with EMT and CRC metastasis.

### Validation of EMT-related protein cluster by PRM

The differential expression levels of ERBB2, GNG2, COL6A1, COL6A2, DCN, COL6A3, LAMB2, TNXB and CAVIN1 were analyzed by LC-MS/MS in the PRM mode (**Figure S2C**). All the quantified peptides for EMT-related proteins exhibited an excellent linear fit between the observed retention time and the iRT in the library. In addition, the retention time and quality of inner-label iRT were stable, and the error of quality was small (**Figures S2D, S2E**). Thus, our method is highly reliable for peptide identification.

The DEPs in cluster (i), including GNG2, COL6A1, COL6A2, DCN, COL6A3, LAMB2, TNXB and CAVIN1, were down-regulated in the Ca and Liver samples compared to P tissues, which was also consistent with the DIA results (**Figure 5E**). In addition, the expression levels of COL6A1, COL6A2, COL6A3 and DCN were higher than that of CAVIN1, GNG2, LAMB2 and TNXB, and all but CAVIN1 were up-regulated in the Liver vs Ca tissues (**Figure 5F**). The peaks of the ERBB2 peptides in PRM demonstrated the superior specificity and stability of our results. (**Figure S3A**), and similar observations were made with CAVIN1, COL6A1, COL6A2, COL6A3, DCN, GNG2, LAMB2 and TNXB (**Figure S3B**). ERBB2 remained the core EMT-related proteins among the 40 DEPs (**Figures 5G**; **S3C**). While ERBB2 was significantly over-expressed in the Ca but down-regulated in the Liver tissues during CRC progression (**Figures 5G**; **S3D**).

### Functional analysis of the core protein and EMT-related protein cluster

**Figure 6:**
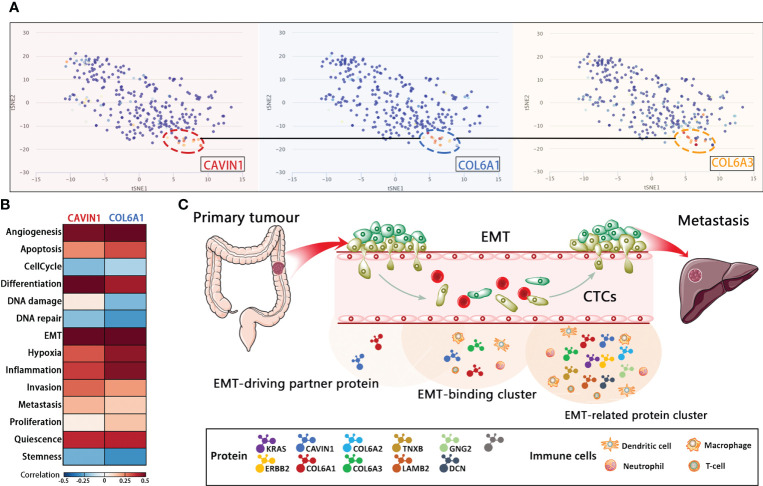
Functional analysis of the core protein and EMT-related protein cluster. **(A)** T-SNE describes the distribution of CRC cells. **(B)** Correlations between EMT-driving partner proteins and functional states in CRC single-cell datasets by CancerSEA analysis. **(C)** Schematic representation of EMT during the metastatic cascade.

The distribution of the target protein was further visualized using t-SNE analysis, which showed that CAVIN1, COL6A1 and COL6A3 were concentrated in the same cell cluster, indicating their involvement in similar malignant processes (**Figure 6A**). After annotating CAVIN1, COL6A1 and COL6A3 as EMT-binding clusters, we analyzed the infiltration level of B cells, CD 8+T cells, CD4+T cells, macrophages, neutrophils and dendritic cells (DCs) in the CRC tumors, and detected significant correlation between the protein expression levels and the number of infiltrating CD4+ T cells, macrophages, neutrophils and DCs from Tumor Immune Estimation Resource (http://timer.comp-genomics.org/) (**Figure S4A**), GNG2, COL6A2, DCN, LAMB2, TNXB, CAVIN1 and COL6A1 have same tendency in infiltration level of B cells, CD 8+T cells, CD4+T cells, macrophages, neutrophils and dendritic cells (DCs) in the CRC tumors (**Figure S4C**). Our results suggest that infiltration of the above immune cells likely promote EMT in CRC. According to the CancerSEA database, the function of CAVIN1 (cor=0.59, p ≤ 0.001) and COL6A1 (cor=0.58, p ≤ 0.01) at the single cell level were significantly related to EMT (**Figure S4B**). Since CAVIN1 and COL6A1 were also part of the EMT-binding clusters, they can be considered EMT-driving partner proteins (**Figure 6B**). In conclusion, the protein network regulating metastasis may also affect the tumor immune micro-environment (**Figure 6C**).

## Discussion

Our findings suggest that EMT in CTCs is a transient process, and most CTCs in metastatic CRC are highly heterogeneous and co-express both epithelial and mesenchymal biomarkers. Therefore, instead of the conventional classification system, we broadly stratified the CTCs as stable or unstable based on their EMT phenotype to evaluate clinical prognosis. The plasticity of the epithelial and mesenchymal CTC phenotypes contributed to liver metastasis of CRC, and are therefore promising indicators of early metastatic events (18). Previous studies show that CTCs undergoing EMT have superior migration, self-seeding and chemoresistance abilities (19–21). Consistent with this, we found that the unstable hybrid E/M CTCs have enhanced ability to metastasize to the liver, which is consistent with the high degree of epithelial–mesenchymal plasticity observed in this phenotype (22).

The proteins secreted by tumor cells are quickly diluted in the bloodstream by nearly thousand-fold. Tumor markers (CEA, AFP, CA125, CA199 and CA153) do not accurately reflect the disease. Therefore, we used DIA and PRM MS-based workflow to detect biomarkers of early micro-metastases. The DIA data sets were qualitatively and quantitatively mined using the highly specific fragment ion maps in a spectral library (23), which ensures accuracy and high efficiency (24). During liquid phase separation, all fragment ion maps of each target parent ion are recorded by PRM (25). Its advantage lies in the use of ultra-high resolution orbit rap quality analyzer that can separate noise from the real signal (25).

Studies show the existence of rare populations with EMT potential within the tumor that are the source of metastasis (26), and the EMT-related metastatic potential may be present even during cancer initiation (27). Since our data confirmed significant proteomic changes during CRC metastasis, we screened for the differentially expressed EMT-related proteins and constructed EMT-related protein clusters by analyzing the enriched pathways. The largest cluster consisted of core proteins including GNG2, COL6A1, COL6A2, DCN, COL6A3, LAMB2, TNXB and CAVIN1, which were down-regulated in the primary tumor and liver metastatic tissues. This cluster is primarily involved in regulating ECM-receptor interaction, focal adhesion, PI3K-Akt signaling pathway and human papillomavirus infection signaling pathway, and correlated strongly with higher infiltration of CD4+ T cells, macrophages, neutrophils, and dendritic cells. Lower immune cell infiltration and differential activation of specific tumor-intrinsic pathways contribute to immune escape (28), which is consistent with our findings as well. Since EMT-related clusters included both EMT-binding proteins (CAVIN1, COL6A1 and COL6A3) and EMT-driving partner proteins (CAVIN1 and COL6A1), a heterogeneous population of cells with EMT potential may reside within the tumor that drive metastasis. In addition, EMT has also been associated with tumor immune escape *via* activation of key immune checkpoints (29). In this regard, the EMT-driving partner proteins (CAVIN1 and COL6A1) are promising biomarkers and therapeutic targets for early metastasis. One of the limitations of this study is that the number of patient samples included in the study is too small and there is a lack of proteomics validation of large-scale colorectal cancer patient tissue samples. On the other hand, we did not record the long-term follow-up data of the patients included in the study. In addition, proteomics and bioinformatics tools can only provide a threshold of guidance, and we still need to improve experimental validation.

In conclusion, liquid biopsies and proteomics data of both tumor tissues and blood samples indicate that EMT biomarkers are promising prognostic factors of mCRC. The CTCs are a highly heterogenous population, and frequently co-exhibit epithelial and mesenchymal features. The clinical significance of CTCs depends on the “stable” or “unstable” phenotype. The EMT-related clusters including receptor proteins (CAVIN1, COL6A1 and COL6A3), interacting proteins (CAVIN1 and COL6A1) and core proteins (ERBB2) influence the distant liver metastatic cascade in CRC. Finally, ERBB2, COL6A1 and CAVIN1 are potential early diagnostic biomarkers of liquid biopsy and therapeutic targets for CRC.

## Data availability statement

The original contributions presented in the study are included in the article/supplementary material. Further inquiries can be directed to the corresponding author.

## Ethics statement

Sample collection and research were in accordance with regulations issued by the National Health Commission of China and the ethical standards formulated in the Helsinki Declaration. Written informed consent was obtained from all patients. The permission for retrospective study was obtained from the institutional review board of Guangxi Medical University Cancer Hospital.

## Author contributions

MH designed the study, led data analyses, and wrote the manuscript. LF and HY obtained clinical information, contributed to the design, experimental work and analysis. DL and LC contributed to the analysis of the study and writing of the manuscript. HR, SM and CW obtained and documented clinical information. XM and WT recruited patients, obtained blood samples and contributed to documentation of clinical information. LY contributed to experimental design, critical discussion of the findings and to the final manuscript. All authors contributed to the article and approved the submitted version.

## Funding

The National Science Foundation (No.82160495). Guangxi University High-level Innovation Team and the Project of Outstanding Scholars Program (2019AC03004) and Guangxi Science and Technology Project (AD19245197). This work was supported by the China Postdoctoral Science Foundation (No. 2019M653812XB).

## Conflict of interest

The authors declare that the research was conducted in the absence of any commercial or financial relationships that could be construed as a potential conflict of interest.

## Publisher’s note

All claims expressed in this article are solely those of the authors and do not necessarily represent those of their affiliated organizations, or those of the publisher, the editors and the reviewers. Any product that may be evaluated in this article, or claim that may be made by its manufacturer, is not guaranteed or endorsed by the publisher.
