# Erratum: Proteomics and liquid biopsy characterization of human EMT-related metastasis in colorectal cancer

**DOI:** 10.3389/fonc.2022.1085628

**Published:** 2022-11-24

**Affiliations:** Frontiers Media SA, Lausanne, Switzerland

**Keywords:** epithelial-mesenchymal transition, proteomics, liquid biopsy, MCRC, CTC (circulation tumor cells)

Due to a production error, the funding statement was erroneously omitted. The correct funding statement is as follows.

The National Science Foundation (No.82160495). Guangxi University High-level Innovation Team and the Project of Outstanding Scholars Program (2019AC03004) and Guangxi Science and Technology Project (AD19245197). This work was supported by the China Postdoctoral Science Foundation (No. 2019M653812XB).

The publisher apologizes for this mistake. The original version of this article has been updated.

