# Supplementary material for: Proteomics and liquid biopsy characterization of human EMT-related metastasis in colorectal cancer
Source: Front Oncol. 2022 Sep 28;12:790096. doi: 10.3389/fonc.2022.790096 (PMC9560976; doi:10.3389/fonc.2022.790096)
Supplement: Supplementary file 1 [file DataSheet_1.docx]

S-Table 1. Distribution of basic clinical features in patients with metastatic colorectal cancer of different EMT subtypes

| Variable | E type | E/M | M type |
| --- | --- | --- | --- |
| Age distribution |  |  |  |
| <60yr | 6 | 33 | 10 |
| ≥60yr | 2 | 14 | 5 |
| Gender |  |  |  |
| Male | 5 | 28 | 9 |
| Female | 3 | 19 | 6 |
| Tumor distribution |  |  |  |
| COAD | 4 | 32 | 10 |
| READ | 4 | 15 | 5 |
| Differentiation |  |  |  |
| High-Mid | 5 | 27 | 10 |
| Low | 3 | 20 | 5 |
| Liver metastases number |  |  |  |
| < 3 | 2 | 17 | 5 |
| ≥ 3 | 6 | 30 | 10 |
| Infiltrating depth |  |  |  |
| T1-T2 | 1 | 9 | 2 |
| T3-T4 | 7 | 38 | 13 |
|  |  |  |  |

S-Table 2. The fluorescent probe capture sequence of Canpatrol® system carries out CTC analysis and captures the fluorescent marks of the EMT morphological transformation of CTC formed during the transfer process.

| Gene | Sequence (5’-3’) |
| --- | --- |
| CK8 | CGTACCTTGTCTATGAAGGAACTTGGTCTCCAGCATCTTGCCTAAGGTTGTTGATGTAGCCTGAGGAAGTTGATCTCGTCCAGATGTGTCCGAGATCTGGTGACCTCAGCAATGATGCTG |
| CK18 | AGAAAGGACAGGACTCAGGCGAGTGGTGAAGCTCATGCTGTCAGGTCCTCGATGATCTTGCAATCTGCAGAACGATGCGGAAGTCATCAGCAGCAAGACGCTGCAGTCGTGTGATATTGG |
| CK19 | CTGTAGGAAGTCATGGCGAGAAGTCATCTGCAGCCAGACGCTGTTCCGTCTCAAACTTGGTTCTTCTTCAGGTAGGCCAGCTCAGCGTACTGATTTCCTCGTGAACCAGGCTTCAGCATC |
| EPCAMP | TGGTGCTCGTTGATGAGTCAAGCCAGCTTTGAGCAAATGAAAAGCCCATCATTGTTCTGGCTCTCATCGCAGTCAGGATCTCCTTGTCTGTTCTTCTGACCTCAGAGCAGGTTATTTCAG |
| Twist: | ACAATGACATCTAGGTCTCCCTGGTAGAGGAAGTCGATGTCAACTGTTCAGACTTCTATCCCTCTTGAGAATGCATGCATTTTCAGTGGCTGATTGGCACTTACCATGGGTCCTCAATAA |
| Vimentin | GAGCGAGAGTGGCAGAGGACCTTTGTCGTTGGTTAGCTGGCATATTGCTGACGTACGTCAGAGCGCCCCTAAGTTTTTAAAAGATTGCAGGGTGTTTTCGGGCCAATAGTGTCTTGGTAG |

**S-Table 3 DEPs in 3 group**

| **Group** |  | **Accession** | **Protein Descriptions** | **Genes** | **P-value** | **log2(FC)** | **FC** |
| --- | --- | --- | --- | --- | --- | --- | --- |
| **P-Liver** |  | P61981 | 14-3-3 protein gamma | YWHAG | 0.04837044 | -0.3536454 | 0.78260412 |
|  |  | Q9HB19 | Pleckstrin homology domain-containing family A member 2 | PLEKHA2 | 0.04964069 | -0.3344951 | 0.79306162 |
|  |  | Q9UJZ1 | Stomatin-like protein 2, mitochondrial | STOML2 | 0.01557449 | -0.3121591 | 0.80543545 |
|  |  | P25786 | Proteasome subunit alpha type-1 | PSMA1 | 0.03543295 | -0.2865902 | 0.81983747 |
|  |  | P05388 | 60S acidic ribosomal protein P0 | RPLP0 | 0.01316079 | -0.2650014 | 0.83219791 |
|  |  | Q9HC84 | Mucin-5B | MUC5B | 0.00024848 | 6.75944858 | 108.341982 |
|  |  | Q13361 | Microfibrillar-associated protein 5 | MFAP5 | 0.00100573 | 6.7806132 | 109.943096 |
|  |  | Q02817 | Mucin-2 | MUC2 | 0.00063923 | 6.97120644 | 125.470678 |
|  |  | Q9Y6R7 | IgGFc-binding protein | FCGBP | 0.00069395 | 7.93537264 | 244.78522 |
|  |  | A8K7I4 | Calcium-activated chloride channel regulator 1 | CLCA1 | 3.5388E-05 | 8.39447066 | 336.501858 |
| **Ca-P** |  | Q6YN16 | Hydroxysteroid dehydrogenase-like protein 2 | HSDL2 | 0.01743211 | -0.3207966 | 0.80062768 |
|  |  | P48960 | CD97 antigen | CD97 | 0.03155451 | -0.315414 | 0.80362036 |
|  |  | P06396 | Gelsolin | GSN | 0.00632453 | -0.3053187 | 0.80926345 |
|  |  | Q6IBS0 | Twinfilin-2 | TWF2 | 0.00308106 | -0.3020085 | 0.81112237 |
|  |  | P61224 | Ras-related protein Rap-1b | RAP1B | 0.04448249 | -0.2966131 | 0.81416152 |
|  |  | Q8WXX5 | DnaJ homolog subfamily C member 9 | DNAJC9 | 2.6696E-05 | 3.57457006 | 11.9138687 |
|  |  | P04626 | Receptor tyrosine-protein kinase erbB-2 | ERBB2 | 0.04994159 | 3.73757007 | 13.3389211 |
|  |  | Q96HR9 | Receptor expression-enhancing protein 6 | REEP6 | 8.3899E-07 | 3.99504401 | 15.9451306 |
|  |  | P25815 | Protein S100-P | S100P | 0.01041157 | 4.34222782 | 20.2834031 |
|  |  | P36952 | Serpin B5 | SERPINB5 | 0.00010587 | 5.37891726 | 41.6116981 |
| **Ca-Liver** |  | Q96HY6 | DDRGK domain-containing protein 1 | DDRGK1 | 0.01464612 | -0.3837267 | 0.76645516 |
|  |  | Q8IUI8 | Cytokine receptor-like factor 3 | CRLF3 | 0.04347054 | -0.3198155 | 0.80117234 |
|  |  | P30101 | Protein disulfide-isomerase A3 | PDIA3 | 0.04263613 | -0.3150196 | 0.80384006 |
|  |  | P53701 | Cytochrome c-type heme lyase | HCCS | 0.00432356 | -0.3002694 | 0.81210075 |
|  |  | Q9P2E9 | Ribosome-binding protein 1 | RRBP1 | 0.03270534 | -0.279566 | 0.82383883 |
|  |  | Q9Y2Y8 | Proteoglycan 3 | PRG3 | 7.8274E-05 | 4.32338101 | 20.0201518 |
|  |  | Q9HC84 | Mucin-5B | MUC5B | 0.00546258 | 4.38534833 | 20.8988019 |
|  |  | Q02817 | Mucin-2 | MUC2 | 0.00495672 | 4.65779872 | 25.2427769 |
|  |  | Q9Y6R7 | IgGFc-binding protein | FCGBP | 0.00876362 | 5.77892337 | 54.9071975 |
|  |  | A8K7I4 | Calcium-activated chloride channel regulator 1 | CLCA1 | 0.0229285 | 6.12830668 | 69.9526437 |

| **Group** | **Accession** | **Protein Descriptions** | **Genes** | **P-value** | **log2(FC)** | **FC** |
| --- | --- | --- | --- | --- | --- | --- |
| **P-Liver** | P12110 | Collagen alpha-2(VI) chain | COL6A2 | 0.01856739 | 1.69342651 | 3.23423949 |
|  | P12109 | Collagen alpha-1(VI) chain | COL6A1 | 0.01186536 | 1.81565269 | 3.52018851 |
|  | P07585 | Decorin | DCN | 0.00867938 | 2.92784228 | 7.60971424 |
|  | Q9HAV0 | Guanine nucleotide-binding protein subunit beta-4 | GNB4 | 0.00632851 | 2.68446268 | 6.42841329 |
|  | P35555 | Fibrillin-1 | FBN1 | 0.02235507 | 2.25683774 | 4.77942724 |
|  | P55268 | Laminin subunit beta-2 | LAMB2 | 0.0291912 | 2.13347239 | 4.38772281 |
|  | Q6NZI2 | Caveolae-associated protein 1 | CAVIN1 | 0.00372102 | 2.1071161 | 4.30829219 |
|  | P04275 | von Willebrand factor | VWF | 0.00805119 | 2.09530548 | 4.27316634 |
|  | P59768 | Guanine nucleotide-binding protein G(I)/G(S)/G(O) subunit gamma-2 | GNG2 | 0.00021779 | 1.71825438 | 3.2903804 |
|  | P12111 | Collagen alpha-3(VI) chain | COL6A3 | 0.03702155 | 1.63955453 | 3.11569611 |
|  | P22105 | Tenascin-X | TNXB | 0.01465321 | 1.2352615 | 2.35424016 |
|  | Q13554 | Calcium/calmodulin-dependent protein kinase type II subunit beta | CAMK2B | 0.00450853 | 1.11055773 | 2.15929107 |
|  | P48729 | Casein kinase I isoform alpha | CSNK1A1 | 0.02198435 | 0.80846833 | 1.75135109 |
|  | Q08209 | Serine/threonine-protein phosphatase 2B catalytic subunit alpha isoform | PPP3CA | 0.01030076 | 0.77359266 | 1.70952162 |
|  | P06213 | Insulin receptor | INSR | 0.00268412 | 0.68872007 | 1.61185288 |
|  | P62879 | Guanine nucleotide-binding protein G(I)/G(S)/G(T) subunit beta-2 | GNB2 | 0.02259912 | 0.58721439 | 1.50234316 |
|  | P62873 | Guanine nucleotide-binding protein G(I)/G(S)/G(T) subunit beta-1 | GNB1 | 0.0325862 | 0.51669889 | 1.43067788 |
|  | P01116 | GTPase KRas | KRAS | 0.03849946 | 0.3209804 | 1.24917915 |
|  | Q9BZK7 | F-box-like/WD repeat-containing protein TBL1XR1 | TBL1XR1 | 0.02823104 | 0.28013326 | 1.21430705 |
|  | P61981 | 14-3-3 protein gamma | YWHAG | 0.04837044 | -0.3536454 | 0.78260412 |
|  | Q9Y265 | RuvB-like 1 | RUVBL1 | 0.0088934 | -0.6241283 | 0.64881168 |
|  | Q00534 | Cyclin-dependent kinase 6 | CDK6 | 0.00034273 | -0.6506689 | 0.63698493 |
|  | P08238 | Heat shock protein HSP 90-beta | HSP90AB1 | 0.04736631 | -0.6830467 | 0.62284854 |
|  | Q13753 | Laminin subunit gamma-2 | LAMC2 | 0.01070964 | -1.7138151 | 0.30485285 |
|  | Q8TDB6 | E3 ubiquitin-protein ligase DTX3L | DTX3L | 0.0186206 | -2.070242 | 0.23811955 |
|  | P01033 | Metalloproteinase inhibitor 1 | TIMP1 | 0.04598185 | -3.6006206 | 0.08243378 |
|  | P35442 | Thrombospondin-2 | THBS2 | 0.00435699 | -4.8146977 | 0.03553297 |
| **Ca-P** | P63104 | 14-3-3 protein zeta/delta | YWHAZ | 0.00445246 | 0.79350566 | 1.73328111 |
|  | P04626 | Receptor tyrosine-protein kinase erbB-2 | ERBB2 | 0.04994159 | 3.73757007 | 13.3389211 |
|  | P01033 | Metalloproteinase inhibitor 1 | TIMP1 | 0.0086764 | 2.03684489 | 4.10347139 |
|  | Q8TDB6 | E3 ubiquitin-protein ligase DTX3L | DTX3L | 0.03665207 | 1.85376884 | 3.61443173 |
|  | Q9Y265 | RuvB-like 1 | RUVBL1 | 0.00011073 | 0.95521689 | 1.93887109 |
|  | P08238 | Heat shock protein HSP 90-beta | HSP90AB1 | 0.0492703 | 0.77892499 | 1.71585185 |
|  | P31946 | 14-3-3 protein beta/alpha | YWHAB | 0.02212687 | 0.56530638 | 1.47970171 |
|  | Q16543 | Hsp90 co-chaperone Cdc37 | CDC37 | 0.02628445 | 0.52934462 | 1.4432734 |
|  | P62753 | 40S ribosomal protein S6 | RPS6 | 0.00222191 | 0.5080116 | 1.42208884 |
|  | Q13547 | Histone deacetylase 1 | HDAC1 | 0.01465123 | 0.45668443 | 1.37238421 |
|  | P67870 | Casein kinase II subunit beta | CSNK2B | 0.00080324 | 0.39973632 | 1.31926677 |
|  | P09619 | Platelet-derived growth factor receptor beta | PDGFRB | 0.01313181 | -0.9750334 | 0.50872808 |
|  | P26006 | Integrin alpha-3 | ITGA3 | 0.02093871 | -1.3586626 | 0.38994361 |
|  | P12111 | Collagen alpha-3(VI) chain | COL6A3 | 0.02663608 | -1.5173139 | 0.34933574 |
|  | P12110 | Collagen alpha-2(VI) chain | COL6A2 | 0.01738169 | -1.599007 | 0.33010411 |
|  | P12109 | Collagen alpha-1(VI) chain | COL6A1 | 0.01611551 | -1.6925379 | 0.3093822 |
|  | P59768 | Guanine nucleotide-binding protein G(I)/G(S)/G(O) subunit gamma-2 | GNG2 | 0.00023754 | -1.7385132 | 0.29967835 |
|  | Q6NZI2 | Caveolae-associated protein 1 | CAVIN1 | 0.02873515 | -1.8065833 | 0.28586715 |
|  | P35558 | Phosphoenolpyruvate carboxykinase, cytosolic [GTP] | PCK1 | 0.03906118 | -2.2261106 | 0.21373416 |
|  | P55268 | Laminin subunit beta-2 | LAMB2 | 0.01172276 | -2.2903454 | 0.20442656 |
|  | P07585 | Decorin | DCN | 0.00926567 | -2.469634 | 0.18053695 |
|  | P22105 | Tenascin-X | TNXB | 0.02155248 | -3.6571128 | 0.07926826 |
| **Ca-Liver** | Q13363 | C-terminal-binding protein 1 | CTBP1 | 0.04893731 | 0.86187974 | 1.81740473 |
|  | Q13573 | SNW domain-containing protein 1 | SNW1 | 0.00097181 | 1.86044545 | 3.63119763 |
|  | P67870 | Casein kinase II subunit beta | CSNK2B | 0.0270764 | 0.50270827 | 1.41687086 |
|  | Q9BZK7 | F-box-like/WD repeat-containing protein TBL1XR1 | TBL1XR1 | 0.00523334 | 0.49855652 | 1.41279929 |
|  | P35558 | Phosphoenolpyruvate carboxykinase, cytosolic [GTP] | PCK1 | 0.04449634 | -2.794427 | 0.14414303 |
|  | P04004 | Vitronectin | VTN | 0.0296544 | -2.0206647 | 0.24644461 |

**Material and Methods**

**Samples and patients collection**

The study was approved by the Guangxi Medical University Ethics Review Board and was performed in agreement with the Helsinki Declaration. All participants provided informed consent. The study was performed at Guangxi Clinical Research Center for Colorectal Cancer (Nanning, China) from March 2018 and May 2019. A total of 70 histologically confirmed mCRC and 30 CRC (total = 100) cases that had matched pairs of tissue and peripheral blood samples for circulating tumor cells (CTCs) analysis, and 12 mCRC cases for which matched pairs of primary tumor tissues, adjacent mucosa tissues and liver metastases tissues were evaluated for proteomics analysis.

**CTCs isolation and identification**

Canpatrol^®^ system (SurExam Bio-Tech, China) be uesd for rapid, size-based capture of CTCs from peripheral blood (PB). CTCs separation, enrichment, and classification identification of two parts, CTC enrichment and multiple RNA in situ analysis and detection.

**CTCs enrichment**

According to the CTC enrichment technology of ISET, using a calibrated membrane with 8uM diameter pores and multiplex mRNA in situ hybridization (ISH) assay to identify and classify CTCs, the optimized enrichment technique is more effective for CTC isolation and characterization. 5ml peripheral blood of patients were collected in EDTA anticoagulated tube, then erythrocytes were removed by a red blood cell lysis buffer (154 mM NH_4_Cl, 10 mM KHCO_3_ and 0.1 mM EDTA). And the cells were transferred to the filtration tube after resuspended in PBS containing 4% formaldehyde for 5 minutes. Then the pump valve was switched on to reach at least 0.08MPa and the manifold vacuum plate valve was then switched on to fulfill filtration.

**Multiple RNA in situ analysis and detection**

To help us distinguish epithelial, mesenchymal and hybrid CTCs. On the membrane of the 24-well plate, cells were treated with a protease before hybridization with indicated capture probe specific for EpCAM, CK8/18/19, vimentin, twist, and CD45(Table 1). After incubation at 42°C for 2 h, cells were washed with buffer to remove the unbound probes. Then cells were incubated with preamplifier solution [30% horse serum, 1.5% sodium dodecyl sulfate, 3mM Tris-HCl (pH 8.0) and 0.5 fmol of preamplifier; the sequences are shown in Table 2] at 42°C for 2 h for the purpose of signal amplification. The membranes were washed with 1000 μl of wash buffer (0.1 × SSC), and then incubated with 100 μl of amplifier solution [30% horse serum, 1.5% sodium dodecyl sulfate, 3mM Tris-HCl (pH 8.0) and 1 fmol of amplifier; the sequences are shown in Table 2]. Fluorescently labeled probes, which had been conjugated with fluorescent dyes Alexa Fluor 594 (for the epithelial biomarkers EpCAM and CK8/18/19), Alexa Fluor 488 (for the mesenchymal biomarkers vimentin and twist), Alexa Fluor 750(for CD45), Alexa Fluor 647 (CD133), were added and incubated at 42°C for 2 min. After staining with DAPI, cells were analyzed with a fluorescence microscope (Olympus BX53, Tokyo, Japan). Red and green fluorescence signal points represent the expression of epithelial and interstitial genes on CTC（Figures.1）.

**
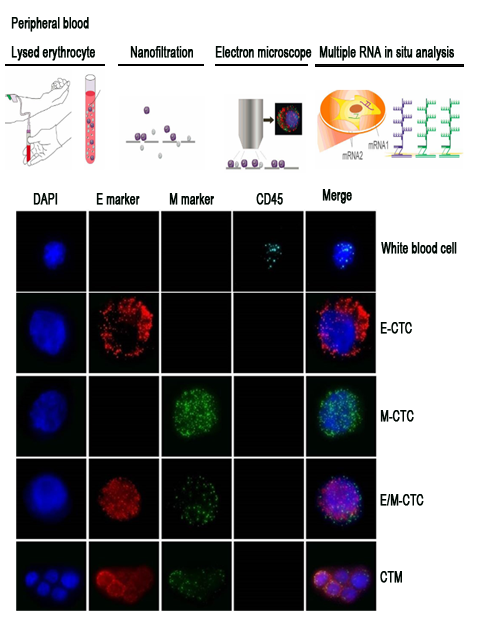
**

Fig.1 CTCs isolation and identification

**Data independent acquisition (DIA)**


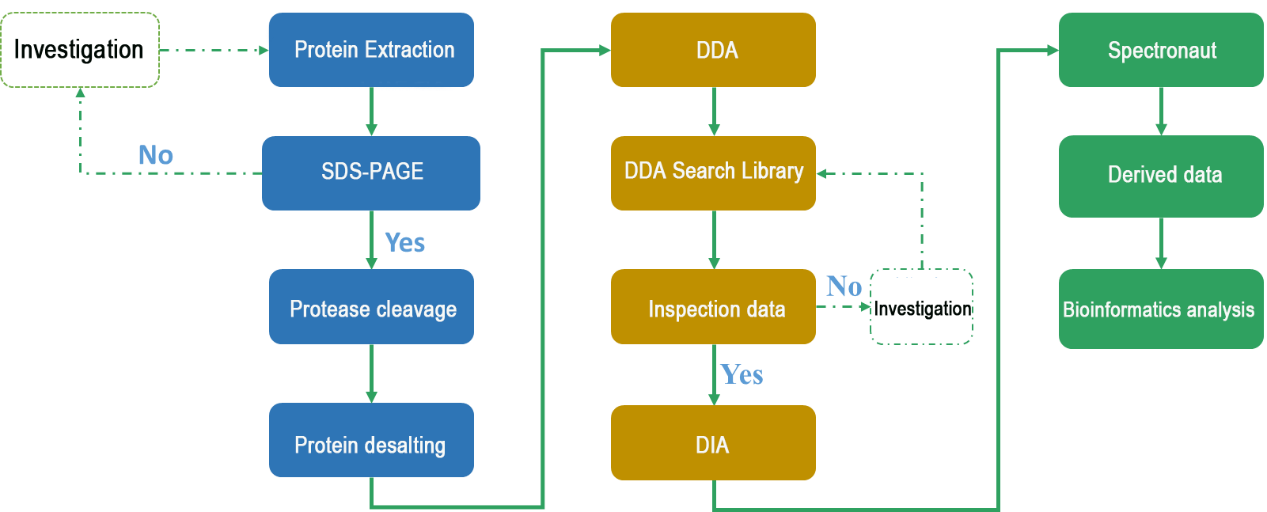


Fig.2 DIA experimental procedure

**Sample preparation**

Frozen samples were transferred into low protein binding tubes (1.5ml Eppendorf) and lysed with 300 μL lysis buffer supplemented with1mM PMSF. Then samples were further lysed with sonication. The parameters were set as 1s/1s intervals, 3min time and 80W power. After sonication, the samples were centrifuged at 15000 g for 15 min to remove insoluble particles, repeat once to further exclude precipitation. Protein concentration was determined by BCA assay and aliquoted to store at -80°C。

**Protein Concentration Determination**

According to the instructions of BCA (Bicinchonininc acid) kit, the required volume of BCA working reagent is allocated by buffer A: Buffer B = 50:1 (v/v). Take out part of the protein solution to be tested and dilute it with ultra-pure water (to prevent excessive concentration from exceeding the working range of the standard curve). Prepare a clean 96-well plate and add BSA standard protein solution of gradient as follows: 0, 1, 2, 4, 8, 12, 16, and 20 μL. Then add the corresponding volume of ultra-pure water to each hole to supplement the volume to 20 μL. Two microliter protein solution was added to 96-well plate with three multiple holes for each sample, and the volume of the protein solution was added to 20 μL. Then 200 μL pre-configured working reagent was added into each hole, and the mixture incubate for 30 minutes at the temperature of 37. The absorbance value (wave length 562 nm) was determined by enzyme labeling instrument. Then calculating the standard curve according to the known concentration and absorbance value of the standard protein solution, and substituting the absorbance value of the sample to be measured, the protein concentration value can be calculated.

**SDS-PAGE electrophoresis**

The 10 ug proteins of each sample were acquired and separated by 12% SDS-PAGE gel. Then separation gel was stained by CBB according to Candiano’s protocol: Firstly, the gel was fixed for 2 h and stained for 12 h. After staining, the gel was washed with water until the bands were visualized. Finally, the stained gel was scanned by ImageScanner (GE Healthcare, USA) at the resolution of 300dpi.

**Proteolysis**

According to the measured protein concentration, take the same quality protein from each sample, and dilute different groups of samples to the same concentration and volume. Add 25mm DTT of corresponding volume into the above protein solution to make the DTT final concentration about 5mM, and incubate at 55°C for 30-60min. Then add the corresponding volume of iodoacetamide so that the final concentration is about 10mm, and place in the dark for 15-30min at room temperature. Then 6 times of the volume of precooled acetone in the above system to precipitate the protein, and place it at - 20 °Cfor more than four hours or overnight. After precipitation, take out the sample and centrifuge at 8000g for 10 min at 4 °C for collecting the precipitate. According to the amount of protein, add the corresponding volume of enzymolysis diluent (protein:enzyme =50:1(m/m),100ug of protein add 2ug of enzyme) to redissolve the protein precipitate, then the solutions were incubated for digestion at 37°C for 12h.

**Desalination**

The solutions were added 2-4μL H3PO4 to each sample to adjust the pH value to 1-3.

The digested peptides were desalted by C18-Reverse-Phase SPE Column. Firstly, rinse once with methanol 1mL, once with 90% acetonitrile-water 1mL , once with water ，All solutions contain 0.1% TFA. And the samples were loaded on the column 3 times. Then the column was washed by 0.1%TFA/H2O 3 times. Finally, the peptides was eluted with 90% ACN/H2O (containing 0.1%TFA) 3 times and were lyophilized.

**LC-MS/MS high resolution mass spectrometry**

**RPLC analysis**

RP separation was performed on an 1100 HPLC System (Agilent) using an Agilent Zorbax Extend RP column (5 μm, 150 mm × 2.1 mm). Mobile phases A (2% acetonitrile in HPLC water) and B (90% acetonitrile in HPLC water) were used for RP gradient. The solvent gradient was set as follows: 0~10 min, 98% A; 10~10.01 min, 98%~95% A; 10.01~37 min, 95%~80% A; 37~48 min, 80~60% A; 48~48.01 min, 60~10% A; 48.01~58 min, 10% A; 58~58.01 min, 10~98% A; 58.01~63 min, 98% A. Tryptic peptides were separated at an fluent flow rate of 250μL/min and monitored at 210 nm. Samples were collected for 10-50 minutes, and eluent was collected in centrifugal tube 1-10 every minute in turn. Samples were recycled in this order until the end of gradient. The separated peptides were lyophilized for mass spectrometry.

**Mass spectrometry analysis**

All analyses were performed by a Q-Exactive HF mass spectrometer (Thermo, USA) equipped with a Nanospray Flex source (Thermo, USA). Samples were loaded and separated by a C18 column (50 cm × 75 μm) on an EASY-nLCTM 1200 system (Thermo, USA). The flow rate was 300 nL/min and linear gradient was 90 min (0~82min, 5-44%B; 82~84 min, 44-90% B; 84~90 min, 90% B; mobile phase A = 0.1% FA in water and B = 0.1% FA in 80%ACN).

**DDA**

Full MS scans were acquired in the mass range of 350 – 1650 m/z with a mass resolution of 120000 and the AGC target value was set at 3e6. The 20 most intense peaks in MS were fragmented with higher-energy collisional dissociation (HCD) with collision energy of 27. MS/MS spectra were obtained with a resolution of 30000 with an AGC target of 2e5 and a max injection time of 80 ms. The Q Exactive HF dynamic exclusion was set for 40.0s and run under positive mode.

**DIA**

Full MS scans were acquired in the mass range of 350 - 1250 m/z with a mass resolution of 120000 and the AGC target value was set at 3e6. The 32 acquisition windows in MS were fragmented with higher-energy collisional dissociation (HCD) with collision energy of 28 and each acquisition window has 26 m/z. MS/MS spectra were obtained with a resolution of 30000 with an AGC target of 1e6 and a max injection time is set to auto and run under positive mode.

**Database search**

Spectronaut was used to search all of the raw data thoroughly against the sample protein database. Database search was performed with Trypsin digestion specificity. Alkylation on cysteine was considered as fixed modifications in the database searching. Protein, peptide and PSM’s false discovery rate (FDR) all set to 0.01. For DIA data, the quantification FDR also set to 0.05. Quantity MS-level was set at MS2.

**Parallel reaction monitoring (PRM)**

**Reversed-phase high performance liquid chromatography (RP-HPLC)**

RP separation was performed on an 1100 HPLC System (Agilent) using an Agilent Zorbax Extend RP column (5 μm, 150 mm×2.1 mm). Mobile phases A (2% acetonitrile in HPLC water) and B (90% acetonitrile in HPLC water) were used for RP gradient. The solvent gradient was set as follows: 0-10 min, 98% A; 10-10.01 min, 98%-95% A; 10.01-37 min, 95%-80% A; 37-48 min, 80-60% A; 48-48.01 min, 60-10% A; 48.01-58 min, 10% A; 58-58.01 min, 10-98% A; 58.01-63 min, 98% A. Tryptic peptides were separated at an fluent flow rate of 250μL/min and monitored at 210 nm. Samples were collected for 10-50 minutes, and eluent was collected in centrifugal tube 1-10 every minute in turn. Samples were recycled in this order until the end of gradient. The separated peptides were lyophilized for mass spectrometry.

**PRM Pre-scan**

The sample used for prescanning is equal mix of all samples to correct the retention time of the peptide segment, as the retention time of the entire liquid mass system may shift after a period, so pre-scanning correction must be carried out.

(1) Pre-sweep method setting

A target protein list is imported into SpectroDive and matched to the corresponding Library (i.e. the above DDA Library), the software will automatically calculate the mass/charge ratio of the theoretical peptide sequence of the target protein, and retain unique peptides that satisfy the following conditions:

I: ProteinGroup specific or Proteoctypic specific (which one is selected depends on the degree of redundancy of the FastA library used. If the degree of redundancy is very low, the latter is selected.)

II: Miss cut to zero

III: Immutable modification

IV: Charge number: 2-3

1. Export the above list and set up a mix pre-scan on the mass spectrometry method.
2. Import the pre-scanned RAW data into SpectrDive and correct the retention time according to the iRT standard peptide sections.

(4) Set the scheduled method and export.

**PRM mode detection**

The chromatographic conditions

Mobile phase A: ACN-H2O-FA (0:10, 0:0.1, V/V/V);

Mobile phase B: ACN-H2O-FA (80:20:0.1, V/V/V);

Flow rate: 300 nL/min;

Gradient elution conditions: 0~ 78min, 2-40% B; 78~80 min, 40-90% B; 80~90 min, 90% B.

**Mass spectrometry conditions:**

Import the pre-scan method into the Inclusion list of the Xcalibur-PRM method editing module. Main scanning parameters include: MS2 resolution 17500, Isolation Window 1.m /z, AGC setting as 1e5, maximum accumulated time of 100 ms, HCD energy of 28.

**Bioinformatics analysis process**

Trusted proteins and differential proteins were screened, and then functional analysis was performed on the differential proteins, including GO analysis, Pathway analysis, and protein interaction. Meanwhile, correlation analysis was performed on the differential comparison data, expression pattern clustering heat map, Venn analysis, etc. In addition, key proteins and their functions or pathways should be selected according to the data and the relevant or interested parts to conduct subsequent key research and verification.


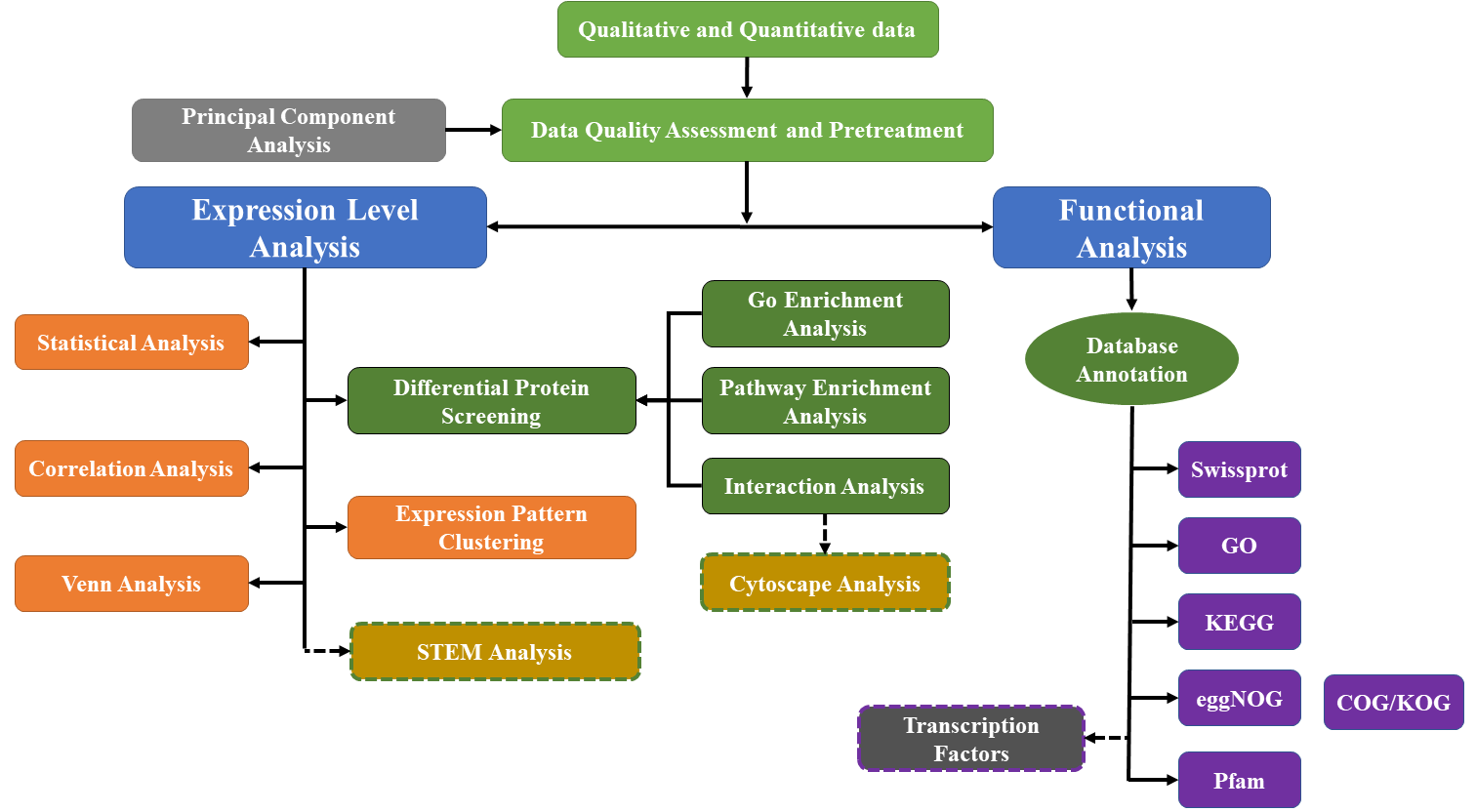


Fig.3 Bioinformatics analysis process

**Combined liquid and mass conditions**

Mass spectrometry scanning parameters:


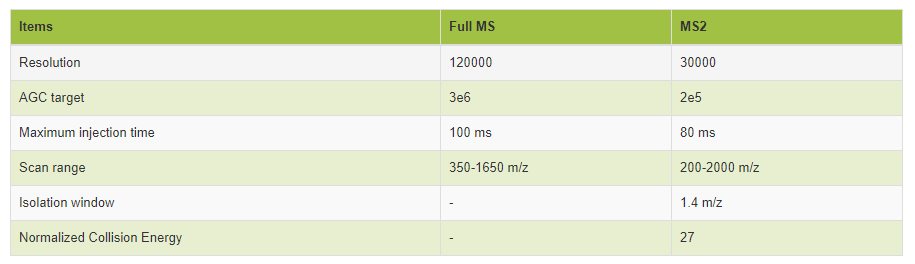


Liquid elution gradient:

The flow rate was 300nL/min. Buffer A was 0.1%FA aqueous solution and buffer B was 0.1%FA /80% ACN/20% water.


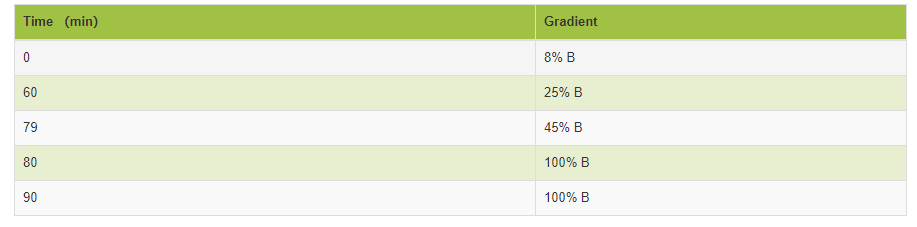


The peptide segment after enzymatic hydrolysis of each sample was separately collected on the machine. The scanning range was set to 350-1250 m/z, and the isolation window was 26 m/z.


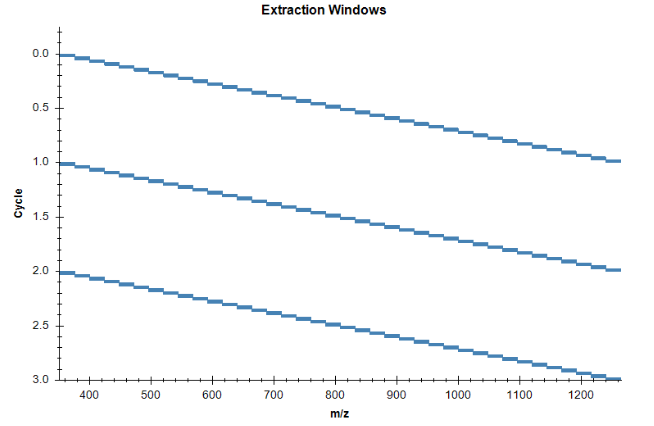


DIA mass spectrometry scanning parameters:


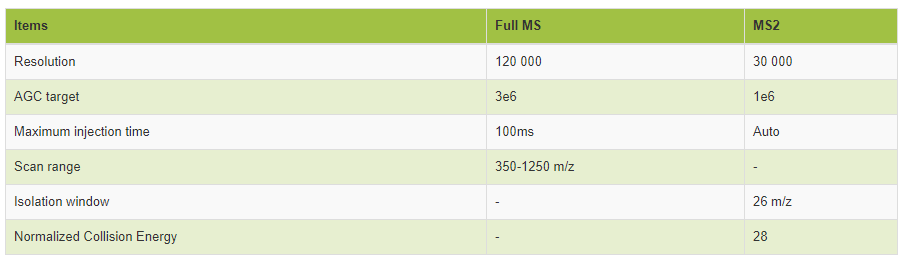


The elution gradient of DIA liquid phase:

The flow rate was 300nL/min. Buffer A was 0.1%FA aqueous solution and buffer B was 0.1%FA /80% ACN/20% water.


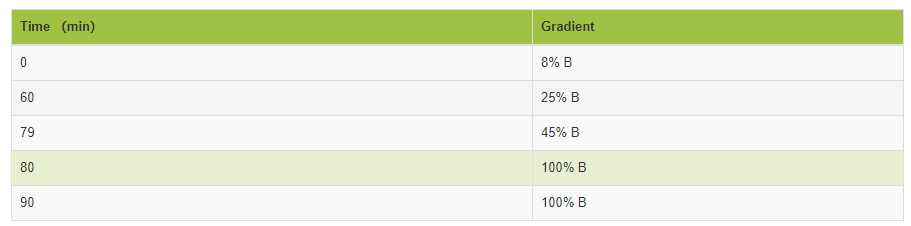


ectronaut Pulsar software to search and build the library. The main parameters are:


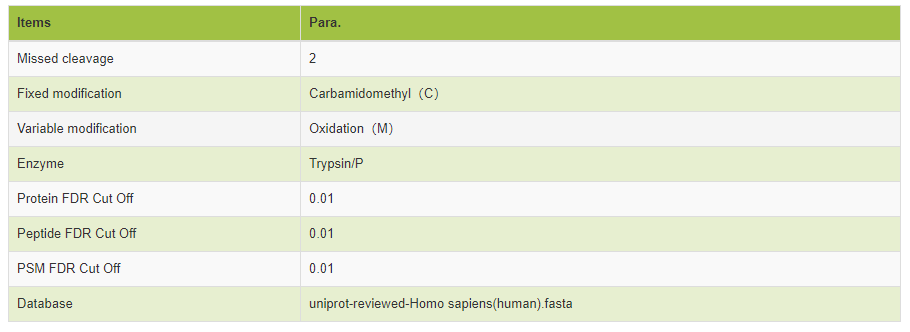


The DIA raw data is processed using the Spectronaut Pulsar software. The key steps are as follows.

1) Open the Analysis module of the Spectronaut Pulsar software and select "+" to create new Analysis;

2) Complete parameter setting step by step and start analysis according to software prompts;

3) After completion of analysis, enter the "Report" module to export quantitative data.


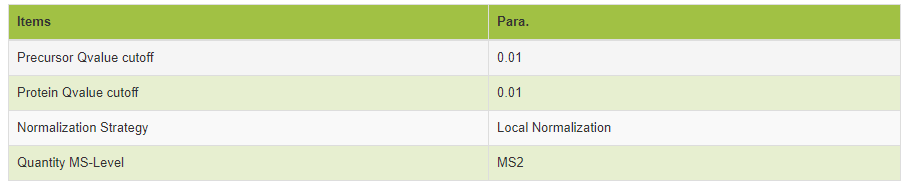


**PRM method export**

1. Import the pre-scanned RAW data into SpectrDive and correct the retention time according to the iRT standard peptide sections. The correction curve is as follows (IRT pre-sweep correction curve):


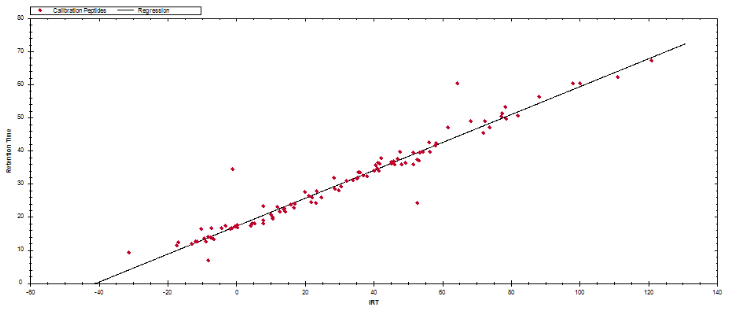


(2) Set the scheduled method and export:

Scan time window setting:


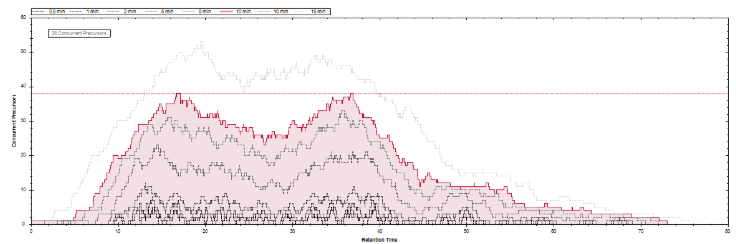


**Reagents and Instruments**

| **Reagent** | **Manufacturer** | **Model** |
| --- | --- | --- |
| TMT10 labeling kit | ThermoScientific | 90111LCS |
| TMT6 labeling kit | ThermoScientific | 90066B |
| iTRAQ Labeling Kit | ABSCIEX | 4381663 |
| Plasma de-abundance protein kit (small column) | ThermoFisher | 85165 |
| Plasma de-abundance protein kit (large column) | Millipore | 122642 |
| SDS lysate | Sangon Biotech | P0013G |
| BCA kit | ThermoScientific | 23227 |
| Mass spectrometry water | ThermoScientific | W6-4 |
| Mass spectrometric grade acetonitrile | ThermoScientific | A955-4 |
| PMSF | Amresco | PB0425-5G |
| Disodium hydrogen phosphate dodecahydrate | Sangon Biotech | ST1725-500G |
| Sodium dihydrogen phosphate monohydrate | Sangon Biotech | S0823-100G |
| NaCl | Sangon Biotech | ST1218-1000G |
| 40%Acr-Bis | Sangon Biotech | B546014-0500 |
| Tris-HCl（pH6.8 pH8.8） | Sangon Biotech | B546020-0250 B546019-0250 |
| APS | Sangon Biotech | AB0072-25G |
| TEMED | Sangon Biotech | A100761-0025 |
| Tris | Sangon Biotech | A600194-0500 |
| Glycerin | Sangon Biotech | G0854-500ML |
| BPB | Sangon Biotech | B0449-5G |
| TFA | Sangon Biotech | TS4295-013 |
| IAA | Sangon Biotech | A600539-0005 |
| Urea | Sangon Biotech | UB0148-2000G |
| TEAB | Sangon Biotech | A510932-0500 |
| DTT | Sangon Biotech | A620058-0005 |
| Glycine | Sangon Biotech | 17-1323-01 |
| SDS | Sangon Biotech | 17-1313-01 |
| Phosphoric acid | Sangon Biotech | 10015418 |
| Pancreatin | Sangon Biotech | HLS TRY001C |
| G-250 | Sigma | 27815-25G-F |
| Absolute ethanol | GENERAL-REAGENT | G73537B |
| Isopropanol | GENERAL-REAGENT | G75885B |
| Tris saturated phenol | Solarbio life sciences | T0250 |
| Formic acid | CNW | CAS:64-18-6 |
| Phosphatase inhibitor | Roch | 4906837001 |
| TiO2 | Sangon Biotech | 5020-75000 |
| Sucrose | Sangon Biotech | A100335-0250 |
| EDTA-2Na | Sangon Biotech | A500838-0500 |
| acetone | Sangon Biotech | 40064485 |
| NaCl | Sangon Biotech | A501218-0001 |
| Ammonium acetate | Sangon Biotech | CAS 631-61-8 |
|  |  |  |
| **Instrument** | **Manufacturer** | **Model** |
| Q ExactiveMC-ICP-MS | ThermoFisher |  |
| Q Exactive PlusMC-ICP-MS | ThermoFisher |  |
| Easy-nLC 1200FPLC | ThermoFisher |  |
| Orbitrap FusionMC-ICP-MS | ThermoFisher |  |
| Q Exactive HFMC-ICP-MS | ThermoFisher |  |
| High speed refrigerated centrifuge | Shanghai Lu Xiangyi Centrifuge Instrument Co., Ltd | TGL-16A |
| SDS-PAGEgel electrophoresis | Beijing Liuyi Instrument Factory | DYY-6C |
| Microplate System | Shanghai Kehua Experimental System Co., Ltd. | ST-360 |
| ImageScanner | EPSON | ES-1000G |
| Precision electronic balance | Shanghai Yueping Scientific Instrument Co., Ltd. | FA2004B |
| freeze dryer | SCIENTZ | SCIENTZ-10N |
| High pH separation liquid chromatograph | Agilent | Agilent 1100 series |
| PolySULPHOETHYL A (5 μm, 200 Å) column (200 × 9.4 mm) | PolyLC Inc. | 209SE0502 |
| 96 well SPE desalting column | ThermoFisher | 60309-001 |
| Ultrasonic Cell Disruptor | SCIENTZ | SCIENTZ-IID |
| 3KDultrafiltration tube | Miilipore | UFL900396 |
