# Supplementary material for: Proteomics and liquid biopsy characterization of human EMT-related metastasis in colorectal cancer
Source: Front Oncol. 2022 Sep 28;12:790096. doi: 10.3389/fonc.2022.790096 (PMC9560976; doi:10.3389/fonc.2022.790096)
Supplement: Supplementary file 2 [file DataSheet_2.zip › Supplementary_Image_Captions.DOCX]

**Supplementary FIGURE 1** (**A**) the expression levels of MSH2, MSH6, PMS2 and MLH1 were analyzed by IHC. (**B**) Patients included in proteomic studies were presented with superficial muscular, perineural and vascular invasion, which are indicative of the early stages of metastasis. (**C**) Principal component analyses and hierarchical clustering analyses revealed distinct protein expression patterns between the paired Ca, P and Liver samples. (**D**) Up-regulated proteins and down-regulated proteins. (**E**) The top 50 enriched pathways. (**F**) Gene ontology (GO) Enrichment analysis of the DEPs further revealed that the most significant biological processes. (**G**) The top 50 DEPs showed significant correlation in each group.

**Supplementary FIGURE 2** (**A**) EMT-related proteins regulate DNA repair, angiogenesis, cell proliferation, apoptosis, glycolysis/gluconeogenesis, protein synthesis, cell cycle and proteolysis. (**B**) Protein-protein interaction (PPI) network was constructed with the EMT-related DEPs. (**C-D**) LC-MS/MS in the PRM mode. (**E**) the retention time and quality of inner-label iRT. (**F**) Shown are proportions of E/M phenotype CTCs in individual mCRC patients (n=70) and CRC patients (n=30)

**Supplementary FIGURE 3** (**A**) The peaks of the ERBB2 peptides in PRM. Each column represents each patient. The first row represents cancer tissue (Ca), the second row represents para cancer tissue (P), and the third row represents liver metastasis tissue (Liver). (**B**) The peaks of the CAVIN1, COL6A1, COL6A2, COL6A3, DCN, GNG2, LAMB2 and TNXB in PRM. (**C**) ERBB2 remained the core EMT-related proteins among the 40 DEPs.

**Supplementary FIGURE 4**  (**A**) correlation between the protein expression levels and the number of infiltrating CD4+ T cells, macrophages, neutrophils and DCs. Using the timer (tumor immune estimation resource) database to analyze the infiltration of immune cells in tumor tissues with high-throughput sequencing (RNA SEQ expression profile) data. (**B**) According to the CancerSEA database, the function of CAVIN1 and COL6A1 at the single cell level were significantly related to EMT. (**C**) GNG2, COL6A2, DCN, LAMB2, TNXB, CAVIN1 and COL6A1 have same tendency in infiltration level of B cells, CD 8+T cells, CD4+T cells, macrophages, neutrophils, and dendritic cells (DCs) in the CRC tumors.

**Supplementary FIGURE 5** Correlation between tumor markers (CEA, AFP, CA125, CA199 and CA153) and tumor size.
