# Supplementary figures and images for: Proteomics and liquid biopsy characterization of human EMT-related metastasis in colorectal cancer
Source: Front Oncol. 2022 Sep 28;12:790096. doi: 10.3389/fonc.2022.790096 (PMC9560976; doi:10.3389/fonc.2022.790096)

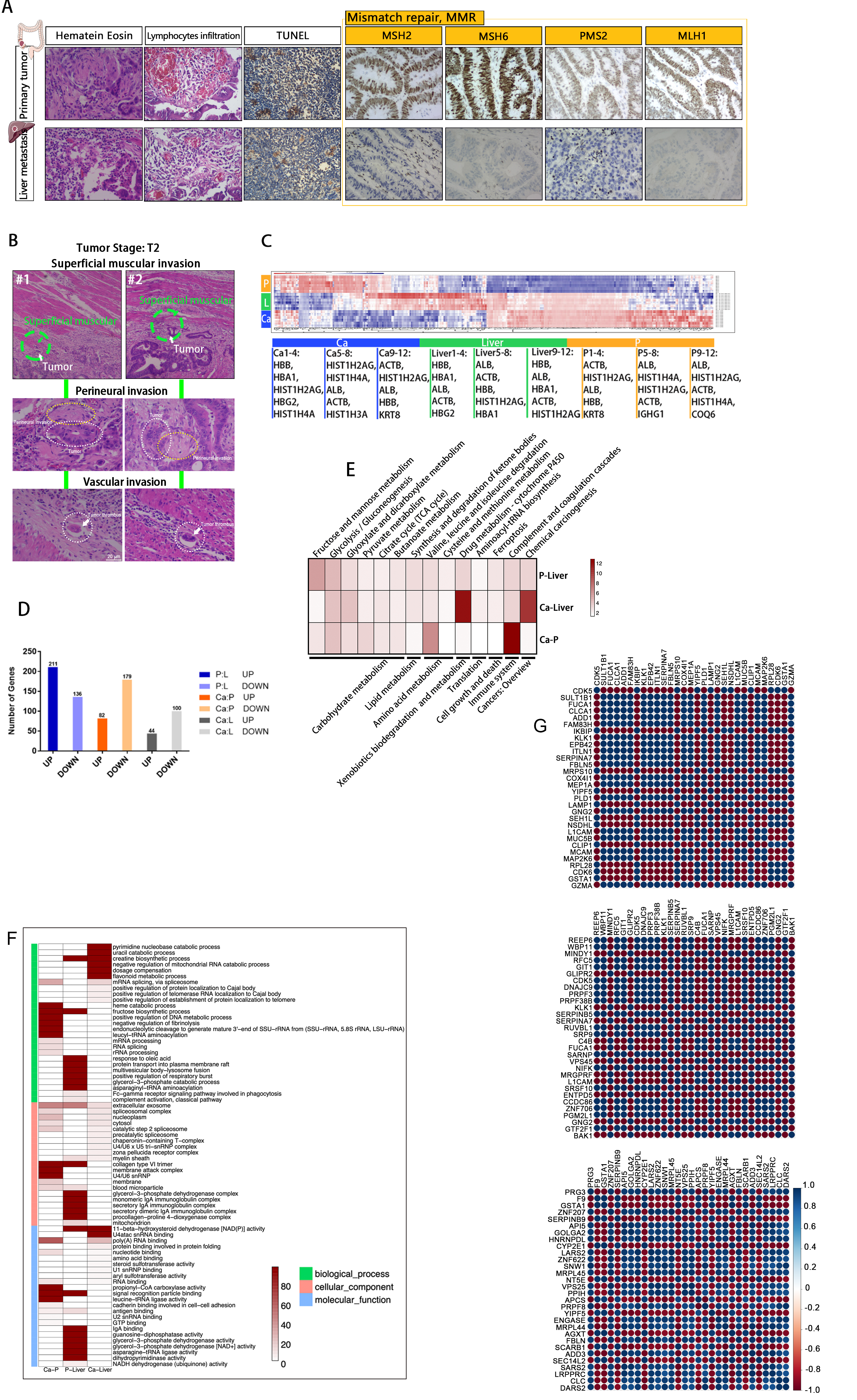

Supplement: Supplementary file 2 [file DataSheet_2.zip › Image 1.TIF]

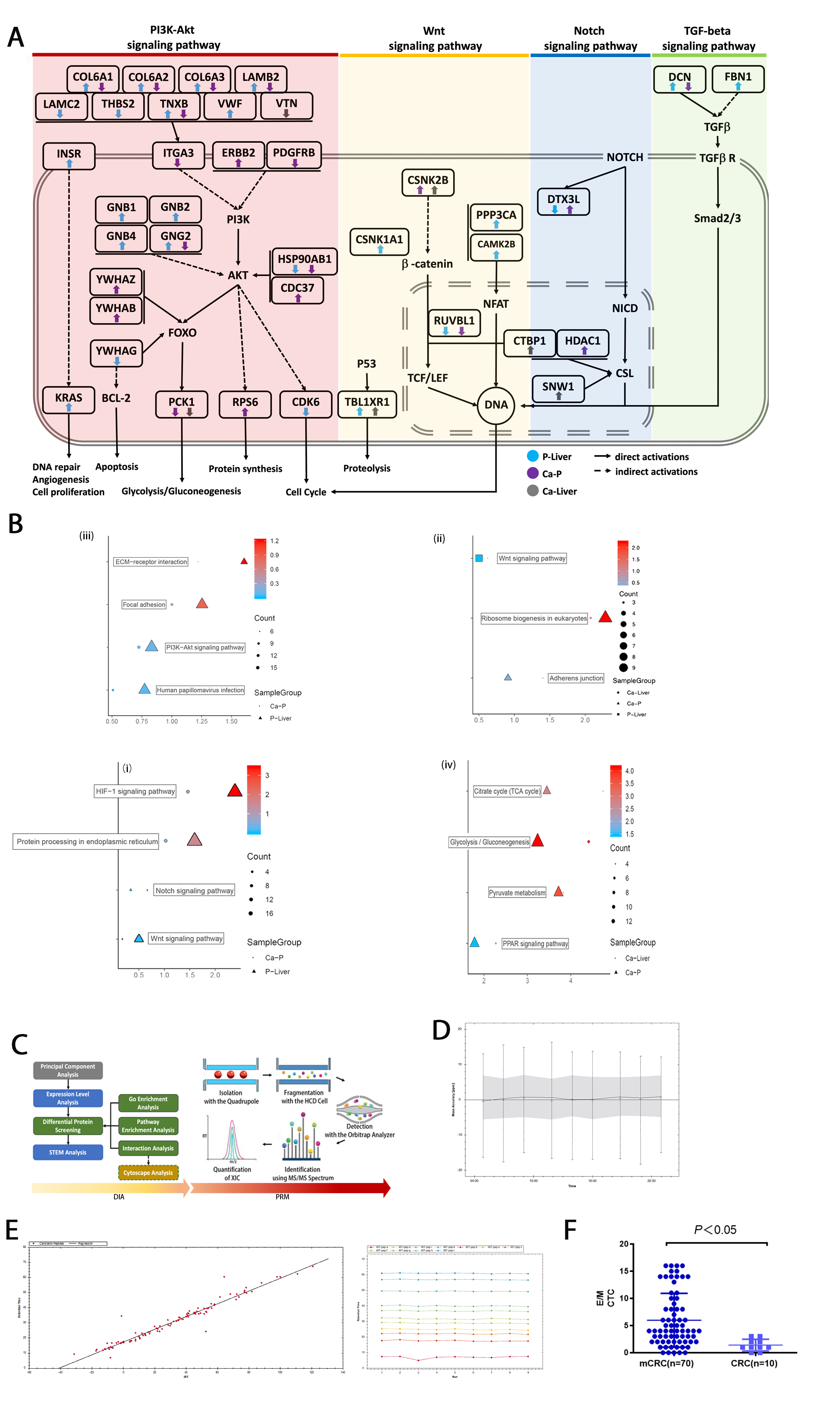

Supplement: Supplementary file 2 [file DataSheet_2.zip › Image 2.TIF]

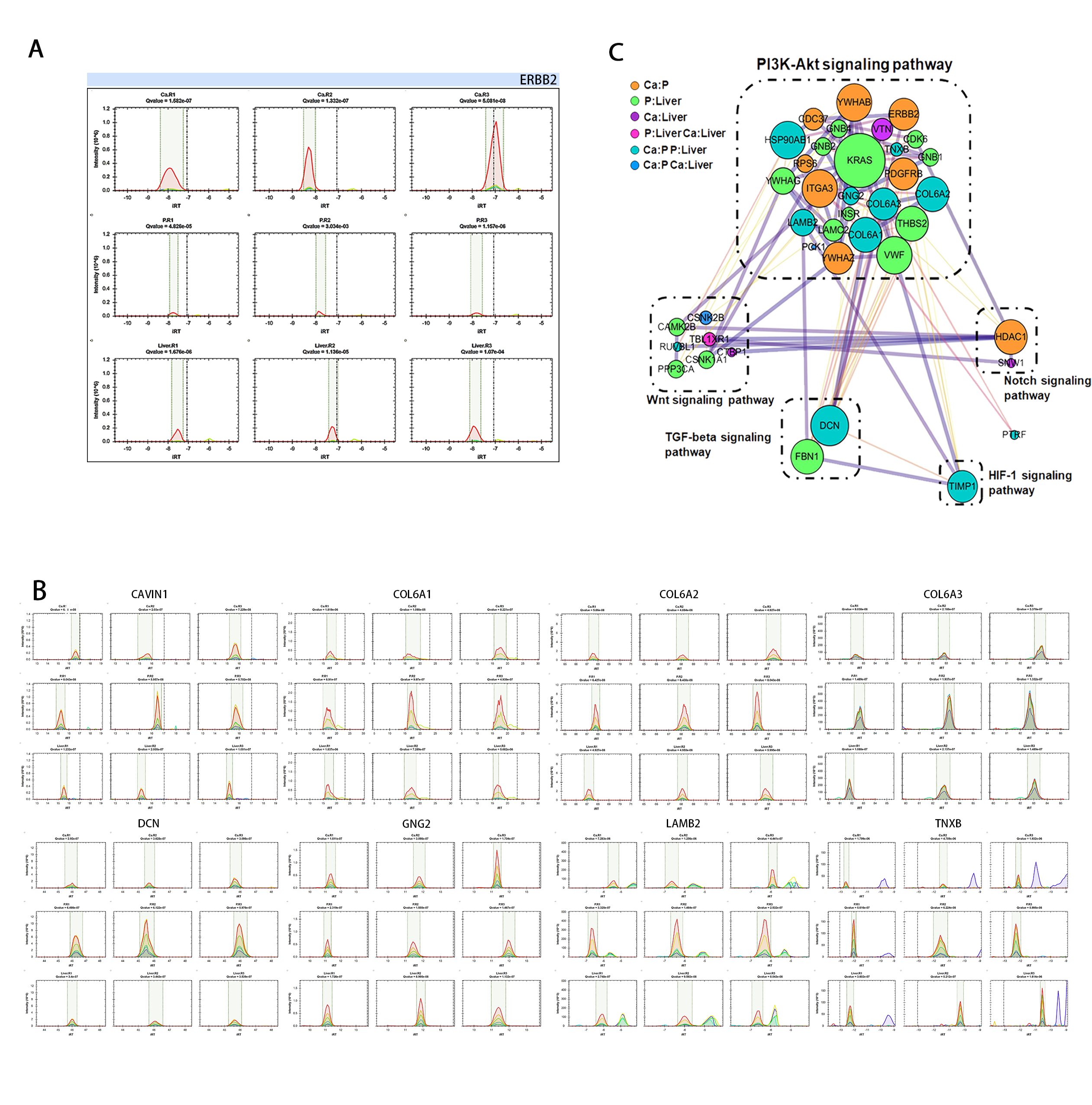

Supplement: Supplementary file 2 [file DataSheet_2.zip › Image 3.TIF]

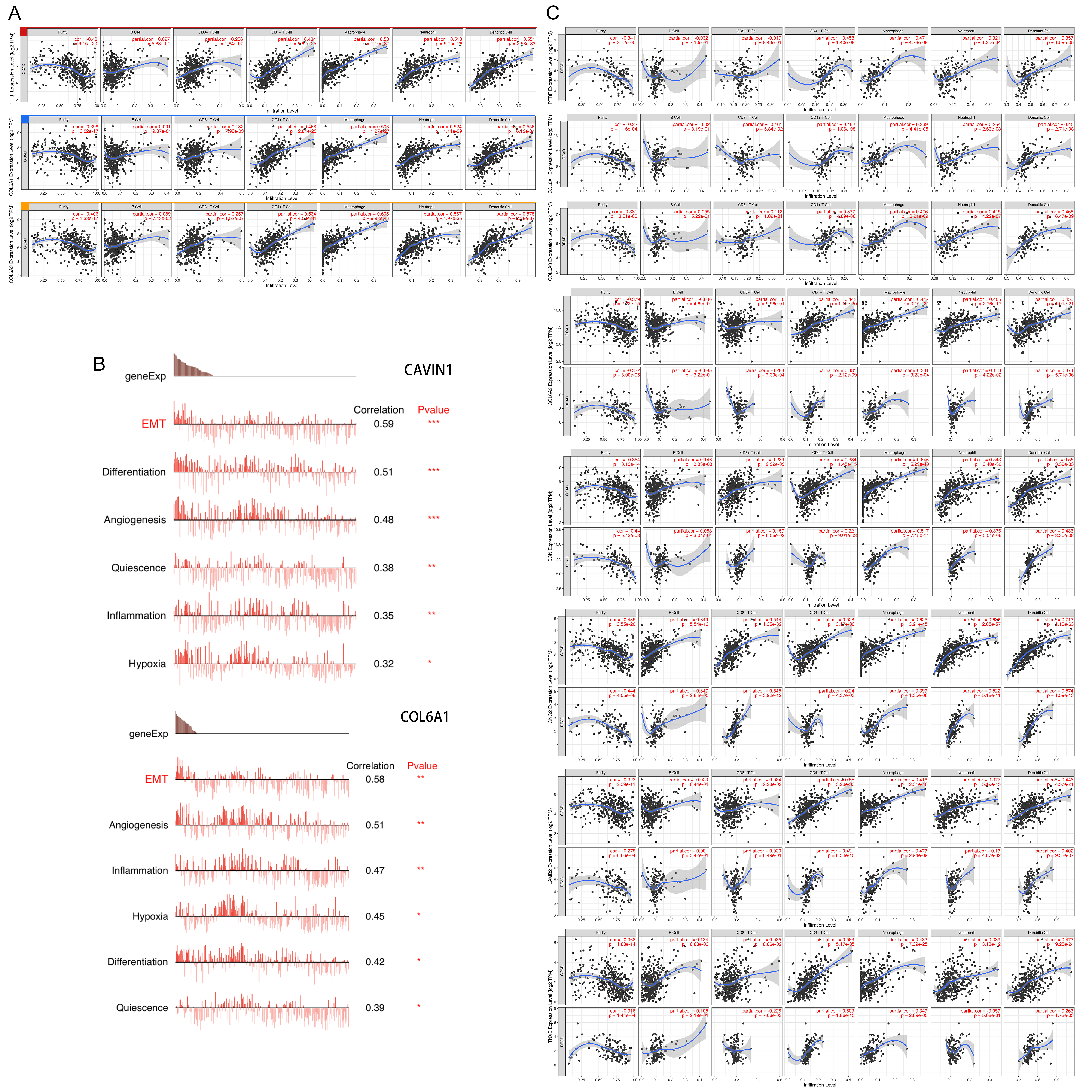

Supplement: Supplementary file 2 [file DataSheet_2.zip › Image 4.TIF]

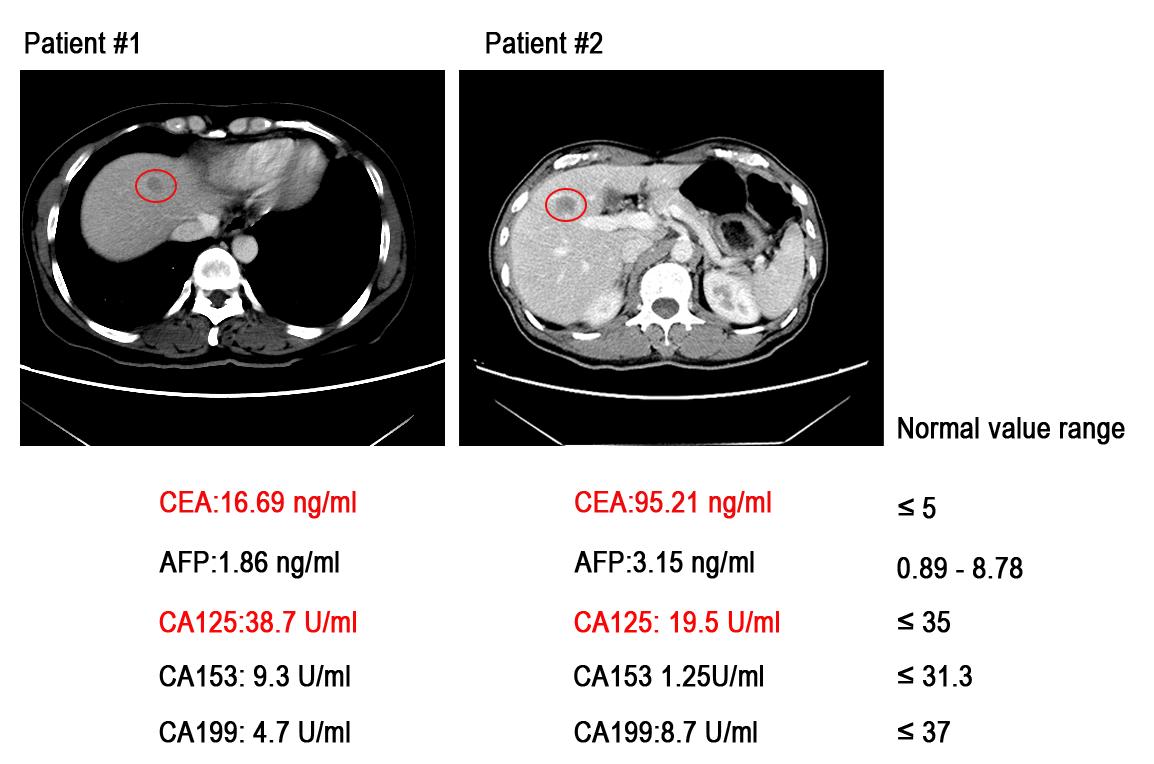

Supplement: Supplementary file 2 [file DataSheet_2.zip › Image 5.TIF]
